# Rapid endothelial cytoskeletal reorganization enables early blood–brain barrier disruption and long-term ischaemic reperfusion brain injury

**DOI:** 10.1038/ncomms10523

**Published:** 2016-01-27

**Authors:** Yejie Shi, Lili Zhang, Hongjian Pu, Leilei Mao, Xiaoming Hu, Xiaoyan Jiang, Na Xu, R. Anne Stetler, Feng Zhang, Xiangrong Liu, Rehana K. Leak, Richard F. Keep, Xunming Ji, Jun Chen

**Affiliations:** 1 Center of Cerebrovascular Disease Research, University of Pittsburgh School of Medicine, Pittsburgh, Pennsylvania 15213, USA; 2 China-America Institute of Neuroscience, Xuanwu Hospital, Capital Medical University, Beijing 100053, China; 3 State Key Laboratory of Medical Neurobiology, Institute of Brain Science and the Collaborative Innovation Center for Brain Science, Fudan University, Shanghai 200032, China; 4 Geriatric Research, Educational and Clinical Center, Veterans Affairs Pittsburgh Health Care System, Pittsburgh, Pennsylvania 15261, USA; 5 Division of Pharmaceutical Sciences, Mylan School of Pharmacy, Duquesne University, Pittsburgh, Pennsylvania 15282, USA; 6 Department of Neurosurgery, University of Michigan, Ann Arbor, Michigan 48109, USA

## Abstract

The mechanism and long-term consequences of early blood–brain barrier (BBB) disruption after cerebral ischaemic/reperfusion (I/R) injury are poorly understood. Here we discover that I/R induces subtle BBB leakage within 30–60 min, likely independent of gelatinase B/MMP-9 activities. The early BBB disruption is caused by the activation of ROCK/MLC signalling, persistent actin polymerization and the disassembly of junctional proteins within microvascular endothelial cells (ECs). Furthermore, the EC alterations facilitate subsequent infiltration of peripheral immune cells, including MMP-9-producing neutrophils/macrophages, resulting in late-onset, irreversible BBB damage. Inactivation of actin depolymerizing factor (ADF) causes sustained actin polymerization in ECs, whereas EC-targeted overexpression of constitutively active mutant ADF reduces actin polymerization and junctional protein disassembly, attenuates both early- and late-onset BBB impairment, and improves long-term histological and neurological outcomes. Thus, we identify a previously unexplored role for early BBB disruption in stroke outcomes, whereby BBB rupture may be a cause rather than a consequence of parenchymal cell injury.

## Abstract (web summary)

Matrix metalloproteinases (MMPs) released from infiltrating immune cells are a major contributor to blood-brain barrier (BBB) breakdown following stroke. Here, the authors identify an early, MMP-independent BBB breakdown mechanism caused by rapid cytoskeletal rearrangements in endothelial cells, which could be inhibited by ADF.

## Graphical abstract

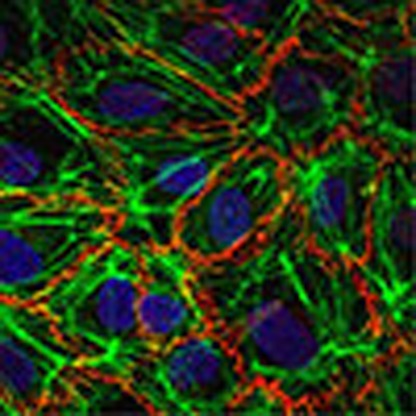

Blood–brain barrier (BBB) dysfunction is a characteristic feature of many neurological conditions, including ischaemic and haemorrhagic stroke, traumatic brain injury, multiple sclerosis, neurodegenerative diseases and brain tumours^1^. However, it is debatable whether BBB dysfunction is the cause or consequence of brain parenchymal injury, partly due to a lack of endothelial cell (EC)-specific interventions. During and after ischaemic strokes, BBB breakdown and the resulting brain oedema are two of the most disabling sequelae and are associated with poor clinical prognosis^2^. The precise mechanism underlying increased BBB permeability in the early stages after cerebral ischaemia/reperfusion (I/R) is poorly understood. However, early BBB permeability may be partially reversible^3^,^4^,^5^, making it a rational target for therapeutic interventions, especially during a post-ischaemic reperfusion event such as under thrombolytic treatment.

The integrity of the BBB is maintained by multiple components, including the tight junction (TJ)-sealed capillary ECs, astrocyte endfeet, pericytes and the extracellular matrix (ECM)^1^. In various tissues, I/R can initiate rapid pathological changes in microvessels that activate the innate immune system and promote endothelial paracellular hyperpermeability^6^,^7^. The early induction of hyperpermeability is usually not accompanied by overt injury, but is associated with subtler alterations, such as a widening of endothelial junctions^7^. Shortly after ischaemic injury, stressed ECs and perivascular astrocytes release a plethora of chemokines and cytokines^6^. Chemical mediators such as interleukins and tumour necrosis factor promote the expression of adhesion molecules on ECs to attract leukocytes to the site of injury, while activated matrix metalloproteinases (MMPs) degrade endothelial junctional proteins (JPs) and the ECM^6^,^8^,^9^. The release of chemokines, cytokines and MMPs, and the upregulation of leukocyte adhesion molecules exacerbate injury in the neurovascular unit^10^,^11^,^12^. Consequently, the impaired BBB permits the infiltration of peripheral immune cells (for example, neutrophils and macrophages) into the brain, bringing with them additional deleterious mediators and resulting in permanent BBB damage in a self-perpetuating loop. Thus, restoring EC structure while simultaneously blocking the detrimental consequences of inflammation may provide a unified and innovative therapeutic strategy for brain protection against I/R injury.

Under physiological conditions, cerebral ECs are fused by intercellular junctions, including TJs and adherens junctions (AJs). The TJ proteins occludin and claudin, and the AJ protein cadherin are anchored to the actin cytoskeleton by multiple accessory proteins (for example, zonula occludens (ZO)-1, ZO-2 and ZO-3)^13^. Dynamic interactions between the cytoskeleton and JPs are therefore important for BBB maintenance^13^,^14^. Following exposure to specific stressors such as hypoxia, free radicals, cytokines and chemokines, the actin that is normally distributed throughout the EC as short filaments and diffuse monomers is polymerized into linear stress fibres across the EC interior^15^. This polymerization is accompanied by actomyosin contraction and increased cytoskeletal tension, resulting in contracted cell morphology, impaired junctional sealing and, eventually, hyperpermeability^16^,^17^,^18^. The underlying mechanisms that modulate EC structure in the face of I/R insults remain understudied and represent an overlooked opportunity to prevent early disturbances in BBB function.

Here we investigate the mechanism underlying early BBB disruption after stroke using a clinically relevant transient focal cerebral ischaemia (tFCI) and reperfusion model. The results suggest that I/R-induced BBB impairment is initiated by subtle cytoskeletal rearrangements in brain ECs, thereby increasing their vulnerability to attack by MMPs from infiltrating immune cells. We hypothesize that stabilizing EC structure can preserve BBB integrity and promote long-term functional recovery after stroke. Importantly, our approach using EC-specific gene manipulation provides evidence that early BBB damage is a cause rather than a result of parenchymal cell injury.

## Results

### tFCI can disrupt the BBB independently of MMPs

**Figure 1:**
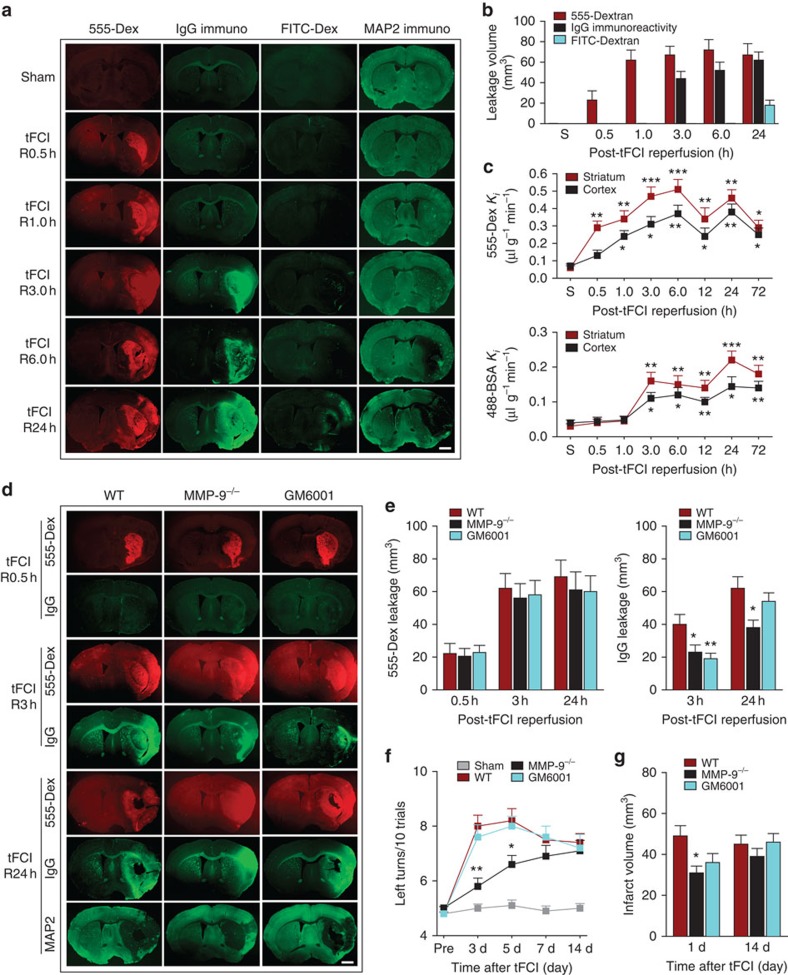
Early-onset and progressive BBB disruption through both MMP-independent and -dependent mechanisms in an *in vivo* stroke model. (**a**) Representative images of coronal brain sections showing the leakage of two fluorescent tracers Alexa555-dextran (3 kDa, red) and FITC-dextran (2,000 kDa, green) into brain parenchyma at indicated reperfusion (R) time points after 1 h of tFCI. Extravasation of endogenous plasma IgG into the central nervous system was visualized on adjacent sections from the same brains by applying fluorescent secondary antibodies against endogenous mouse IgG molecules (green). MAP2 immunostaining was used to illustrate infarcts in the same brains, as observed in the striatum at 6 h and in the entire MCA territory at 24 h. Scale bar, 1 mm. (**b**) Volume of leakage of Alexa555-dextran, endogenous IgG and FITC-dextran in sham-operated brains (S) and at 0.5–24 h of reperfusion after tFCI. *n*=4–5 mice per group. (**c**) In a separate cohort of mice, extravasation of Alexa555-dextran (3 kDa) and Alexa488-BSA (60–70 kDa) into striatal and cortical parenchyma at 0.5–72 h of reperfusion was quantified by calculating their blood–brain transfer coefficient *K*_i_. *n*=5–6 mice per group. *P≤0.05, **P≤0.01, ***P≤0.001 versus sham. (**d**–**g**) tFCI was induced for 1 h in WT or MMP-9^−/−^ mice followed by reperfusion. In separate groups of WT mice, vehicle or the broad-spectrum MMP inhibitor GM6001 was administered as described in Methods. (**d**) Representative images showing the leakage of Alexa555-dextran (3 kDa, red) and plasma IgG (green) into brain parenchyma at 0.5, 3 and 24 h of reperfusion. MAP2 immunostaining illustrates infarction in the same brains. Scale bar, 1 mm. (**e**) Volume of leakage of Alexa555-dextran and endogenous IgGs in the same groups. (**f**) Sensorimotor dysfunction was assessed by the corner test up to 14 d after tFCI and expressed as the number of left body turns made over the course of 10 trials. (**g**) Brain infarct volumes were calculated on MAP2-stained sections at 1 and 14 d after tFCI. *n*=6 mice per group. *P≤0.05, **P≤0.01 versus WT. d, days.

We characterized the spatial distribution and temporal profile of BBB disruption after tFCI by examining the extravasation of injected fluorescent tracers from the peripheral circulation into the brain. A surprisingly early BBB leakage of the small-molecule fluorescent tracer Alexa555-dextran (3 kDa) into the ischaemic striatum was detected within 30 min after tFCI (Fig. 1a,b). The spatial distribution of this BBB leakage extended across the ischaemic cortex and striatum within 1 h after tFCI. Further deterioration was observed at 3 and 6 h after tFCI, with increased brain permeability to both the small tracer and to larger plasma IgG molecules (∼150 kDa). In contrast, leakage of the very-large-molecule tracer fluorescein isothiocyanate (FITC)-dextran (2,000 kDa) was not detected until 24 h after tFCI. In the same set of brains, the brain infarction expanded from the striatum at 6 h after tFCI into the cortex within 24 h, as indicated by loss of the neuronal marker microtubule-associated protein 2 (MAP2). Although loss of MAP2 was considerably delayed compared with the temporal kinetics of BBB leakage, it followed a similar spatial progression, as apparent from comparing the topography of 555-dextran infiltration at 6 and 24 h with MAP2-negative areas on adjacent sections (Fig. 1a). In separate experiments (Fig. 1c), the blood–brain transfer coefficient *K*_i_ of two differently sized tracers (3 and 60–70 kDa) was determined between 0.5 and 72 h after tFCI, and the results were consistent with the above descriptions.

The MMPs, especially MMP-9 (also known as gelatinase B), are well known to mediate ischaemia-induced BBB disruption by degrading endothelial JPs and the basal lamina of capillaries^8^,^19^,^20^. Previous studies have shown that MMP-2 and MMP-9 are increased in the ischaemic core within 1–2 h following focal cerebral ischaemia^21^. Consistent with these observations, IgG leakage was alleviated in MMP-9^−/−^ mice at 3 and 24 h after tFCI (Fig. 1d,e). This effect could not be attributed to differences in regional cerebral blood flow (Supplementary Table 1) or other physiological parameters, indicating that all groups were exposed to the same degree of ischaemic insult. In contrast to the effects on IgG leakage, *MMP-9* gene ablation failed to ameliorate the leakage of the 3 kDa-dextran at 30 min, 3 or 24 h after tFCI. MMP-9^−/−^ mice showed significantly improved sensorimotor function in the corner test 3 and 5 days after tFCI, and exhibited smaller infarct volumes 24 h after tFCI (Fig. 1f,g). However, the beneficial effect of MMP-9 deficiency on sensorimotor function or infarct volumes was lost by 7 and 14 days after tFCI, respectively, suggesting that other MMPs and/or MMP-independent mechanisms may contribute to infarct evolution after prolonged reperfusion. Alternatively, loss of MMP-9 may impair brain repair processes, as MMP-9 is also known to participate in neurovascular remodelling after stroke^22^,^23^.

Similar to the above-mentioned results in MMP-9^−/−^ mice, administration of the MMP inhibitor GM6001 at the onset of post-stroke reperfusion had no effect on leakage of the 3 kDa-dextran through the BBB at 30 min, 3 or 24 h after tFCI (Fig. 1d,e). In contrast to previously reported results^24^,^25^, GM6001 failed to reduce infarct volume or improve neurobehavioral performance up to 14 days after tFCI (Fig. 1f,g). Nevertheless, GM6001 inhibited IgG extravasation 3 h after tFCI, an effect that was lost at 24 h. Repeated administration of GM6001 beyond 3 h after tFCI may be needed to achieve neuroprotective effects in this model.

In summary, these results support the presence of at least two distinct mechanisms that underlie BBB disruption after tFCI. Whereas the late-onset BBB leakage of larger molecules (∼150 kDa) at 3–6 h after tFCI is MMP-9 dependent, a suggested MMP-9-independent mechanism is responsible for the early onset (within 30–60 min after tFCI) but persistent BBB leakage of smaller molecules (≤3 kDa).

### OGD elicits delayed degradation of JPs

**Figure 2:**
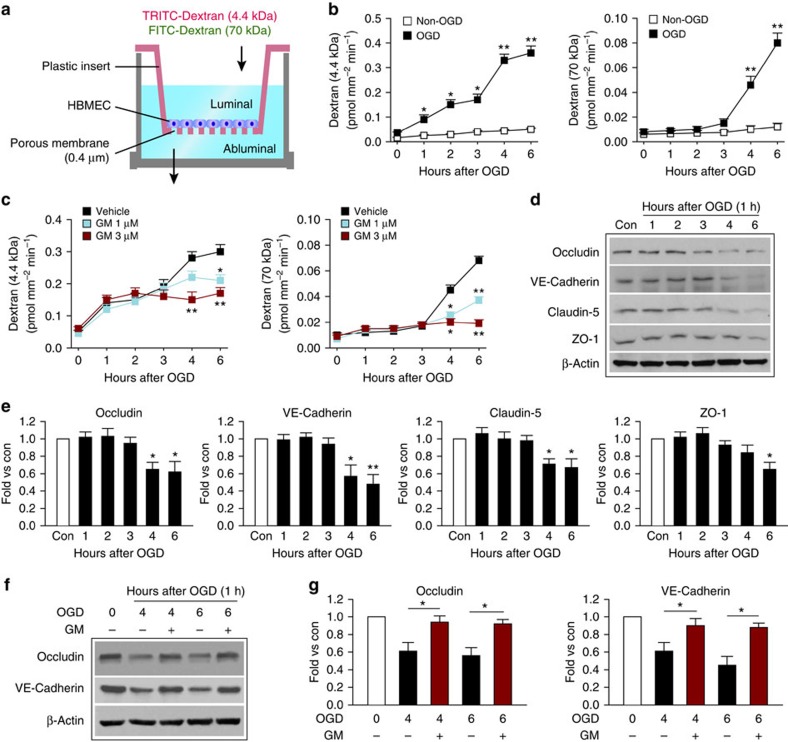
Progressive barrier leakage and delayed degradation of endothelial JPs after an ischaemia-like insult in an *in vitro* BBB model. (**a**) Illustration of the *in vitro* BBB model. An HBMEC monolayer seeded on top of a membrane in the cell culture insert was subjected to 1 h of OGD. Paracellular permeability was determined by measuring the luminal to abluminal diffusion coefficient of a 4.4 kDa TRITC-dextran or a 70 kDa FITC-dextran. (**b**) The diffusion coefficient of the two fluorescent tracers at 0–6 h after OGD or control non-OGD conditions. Data represent four independent experiments. *P≤0.05, **P≤0.01 versus non-OGD. (**c**) The MMP inhibitor GM6001 (1 or 3 μM) or vehicle was applied 30 min before, during and after OGD. The diffusion coefficient of the two tracers was measured 0–6 h after OGD. Data represent four independent experiments. *P≤0.05, **P≤0.01 versus vehicle control. (**d**–**g**) Cultured HBMECs were subjected to 1-h OGD. (**d**,**e**) Expression of TJ proteins occludin, claudin-5 and ZO-1, as well as the AJ protein VE-cadherin was evaluated in HBMECs by western blotting 1–6 h after OGD. β-Actin was used as an internal loading control. Blots were quantified and expressed relative to non-OGD controls (Con). Data represent four independent experiments. *P≤0.05, **P≤0.01 versus Con. (**f**,**g**) Western blots showing that the delayed degradation of occludin and VE-cadherin at 4 and 6 h after OGD was prevented by MMP inhibition with GM6001 (3 μM). Data represent four independent experiments. *P≤0.05 versus vehicle.

To investigate the mechanisms underlying I/R-induced BBB disruption, we adopted an *in vitro* BBB model composed of a monolayer of human brain microvessel endothelial cells (HBMECs; Fig. 2a), and subjected this model to the ischaemia-like insult oxygen–glucose deprivation (OGD) for 1 h. Experiments were performed up to 6 h of reperfusion after OGD, as overt cell death was evident beyond this time (Supplementary Fig. 1). Subtle paracellular hyperpermeability occurred 1–3 h post OGD, with increased diffusion of 4.4 kDa-dextran, but not 70 kDa-dextran across the EC barrier (Fig. 2b*). In vitro* BBB integrity deteriorated progressively with an abrupt increase in luminal to abluminal flux of both tracers at 4 and 6 h post OGD. MMP inhibition with GM6001 dose dependently reduced barrier leakage 4–6 h post OGD (Fig. 2c), but failed to reduce the barrier hyperpermeability to 4.4 kDa-dextran at 1–3 h post OGD.

Western blots revealed that TJ proteins occludin and claudin-5, as well as the AJ protein vascular endothelial (VE)-cadherin were partially degraded in HBMECs at 4–6 h, but not at 1–3 h, post OGD (Fig. 2d,e). The TJ accessory protein ZO-1 was also downregulated at 6 h. This loss of JPs may explain the abrupt increase in barrier permeability observed 4–6 h post OGD. The loss of occludin and VE-cadherin was abolished by MMP inhibition (Fig. 2f,g), consistent with previous findings^8^,^20^. Taken together, these *in vitro* data confirm the role of MMPs in the late phase (⩾4 h) of EC barrier disruption. Similar to the *in vivo* situation, subtle extracellular MMP-independent barrier impairment occurs before the frank loss of JPs and severe barrier damage.

### OGD rapidly induces actin stress fibres in brain endothelia

**Figure 3:**
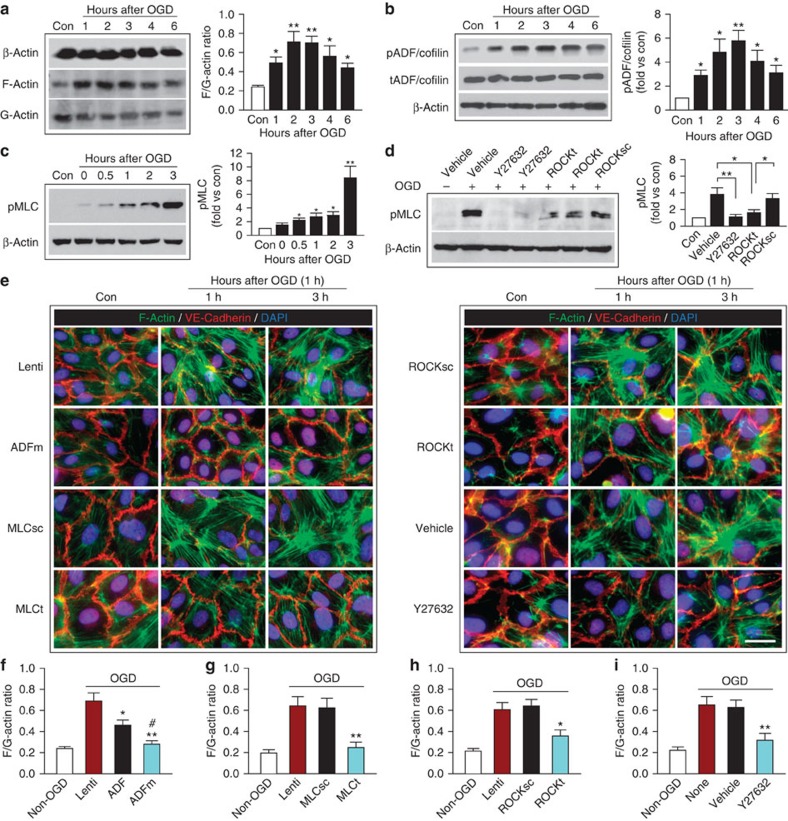
Oxygen–glucose deprivation *in vitro* induces rapid and robust formation of actin stress fibres in endothelial cells. (**a**) HBMECs were exposed to 1-h OGD and the expression of total β-actin, polymerized F-actin and soluble G-actin was examined by western blotting at 1–6 h after OGD. The ratio of F-actin to G-actin in HBMECs was quantified as a measure of stress fibre formation. Data represent four independent experiments. F-actin expression was elevated after OGD, whereas G-actin was downregulated, resulting in an increased F/G-actin ratio. (**b**) Phosphorylation of ADF/cofilin (pADF/cofilin) and expression of total ADF/cofilin (tADF/cofilin) in HBMECs were assessed at 1–6 h after OGD. β-Actin was used as an internal loading control. pADF/cofilin was quantified and expressed relative to non-OGD controls (Con). Data represent four independent experiments. (**c**) Phosphorylation of MLC (pMLC) was evaluated at 0–3 h after OGD by western blotting and expressed relative to non-OGD controls. Data represent four independent experiments. *P≤0.05, **P≤0.01 versus non-OGD. (**d**) HBMECs were treated with vehicle or the ROCK inhibitor Y27632, or infected with lentiviral vectors carrying the scrambled short hairpin RNA (shRNA) sequence (ROCKsc) or ROCK-targeting sequence (ROCKt). Cells were then subjected to 1 h of OGD and pMLC was examined by western blotting at 1 h after OGD. Data represent four independent experiments. *P≤0.05, **P≤0.01. (**e**–**i**) HBMECs were infected with control empty lentivirus (Lenti), or lentiviral vectors carrying HA-tagged WT ADF (ADF), HA-tagged constitutively active mutant ADF (ADFm), MLC-targeting shRNA (MLCt), ROCK-targeting shRNA (ROCKt) or non-targeting scrambled sequences (MLCsc or ROCKsc). In separate cultures, HBMECs were treated with vehicle or Y27632. Cells were subjected to 1-h OGD. (**e**) HBMECs were stained at 1 or 3 h after OGD for F-actin^+^ stress fibres (green) and the AJ protein VE-cadherin (red), and counterstained with 4′,6-diamidino-2-phenylindole (DAPI; blue) for nuclear labelling. Scale bar, 30 μm. OGD-induced stress fibre formation was significantly attenuated by ADFm overexpression, MLC knockdown, ROCK knockdown or ROCK inhibition. (**f**–**i**) The ratio of F-actin to G-actin was quantified at 3 h after OGD. Data represent four independent experiments. (**f**) *P≤0.05, **P≤0.01 versus Lenti. ^#^*P*≤0.05 versus ADF. (**g**) **P≤0.01 versus MLCsc. (**h**) *P≤0.05 versus ROCKsc. (**i**) **P≤0.01 versus vehicle.

In non-central nervous system ECs, stress induces actin polymerization and the formation of stress fibres, which are contractile bundles consisting of crosslinked F-actins. This leads in turn to actomyosin contraction and generates centripetal tension against cell–cell and cell–matrix tethering forces, which increases the permeability of endothelial barriers^26^ and weakens endothelial paracellular connectivity^27^. Considering the essential role of the endothelium in normal BBB integrity, we hypothesized that early BBB breach after I/R results from subtle structural changes in ECs before significant degradation of JPs. The normal balance between assembly and disassembly of actin filaments is regulated by several actin-binding proteins, including the actin depolymerizing factor (ADF)/cofilin family, which is active when dephosphorylated and causes a net depolymerization of actin^28^. In addition, phosphorylation of myosin light chain (MLC) is known to promote the formation of dense stress fibres from short F-actins and to elicit actomyosin contraction^16^,^17^. RhoA and its downstream effector, Rho-associated protein kinase (ROCK), potentiate MLC phosphorylation by inhibiting MLC phosphatase activity^17^,^29^. Our results from cultured HBMECs confirm the participation of these pathways in regulating stress fibre formation after ischaemic insults. OGD induced EC actin cytoskeletal rearrangement by promoting the formation of F-actin from G-actin, a sign of stress fibre formation (Fig. 3a). The F/G-actin ratio in ECs markedly increased as early as 1 h post OGD and remained elevated 6 h post OGD, with no significant change in total β-actin levels. On the one hand, this increase in actin polymerization might be partially explained by OGD-induced phosphorylation and therefore inactivation of ADF/cofilin (Fig. 3b) and the resulting release from endogenous inhibition of actin polymerization. On the other hand, MLC was rapidly phosphorylated at 0–3 h post OGD (Fig. 3c), also serving as a strong driving force for stress fibre formation. ROCK activity contributed to post-OGD MLC phosphorylation, as pharmacological inhibition of ROCK with Y27632 or lentiviral short hairpin RNA knockdown of ROCK (Supplementary Fig. 2) attenuated MLC phosphorylation 1 h post OGD (Fig. 3d).

To further confirm the role of ADF in post-OGD stress fibre formation, we overexpressed hemagglutinin (HA)-tagged wild-type (WT) human ADF or its non-phosphorylatable—thus constitutively active—mutant (ADFm) bearing single-amino-acid substitution (S3A) in HBMECs using lentivirus-mediated transduction (Supplementary Fig. 2). Consistent with the above findings, robust stress fibre formation was visualized by immunostaining for F-actin 1–3 h after OGD (Fig. 3e). Expression of ADFm inhibited actin polymerization and blunted OGD-induced elevation of the F/G-actin ratio, showing an even stronger effect than WT ADF (Fig. 3e,f). Furthermore, short hairpin RNA knockdown of MLC or knockdown/inhibition of ROCK elicited similar results (Fig. 3e,g–i). These results support the view that ADF inhibits OGD-induced stress fibre formation in brain ECs, whereas ROCK/MLC signalling exerts the opposite effect.

### Cytoskeletal and JP reorganization after OGD

**Figure 4:**
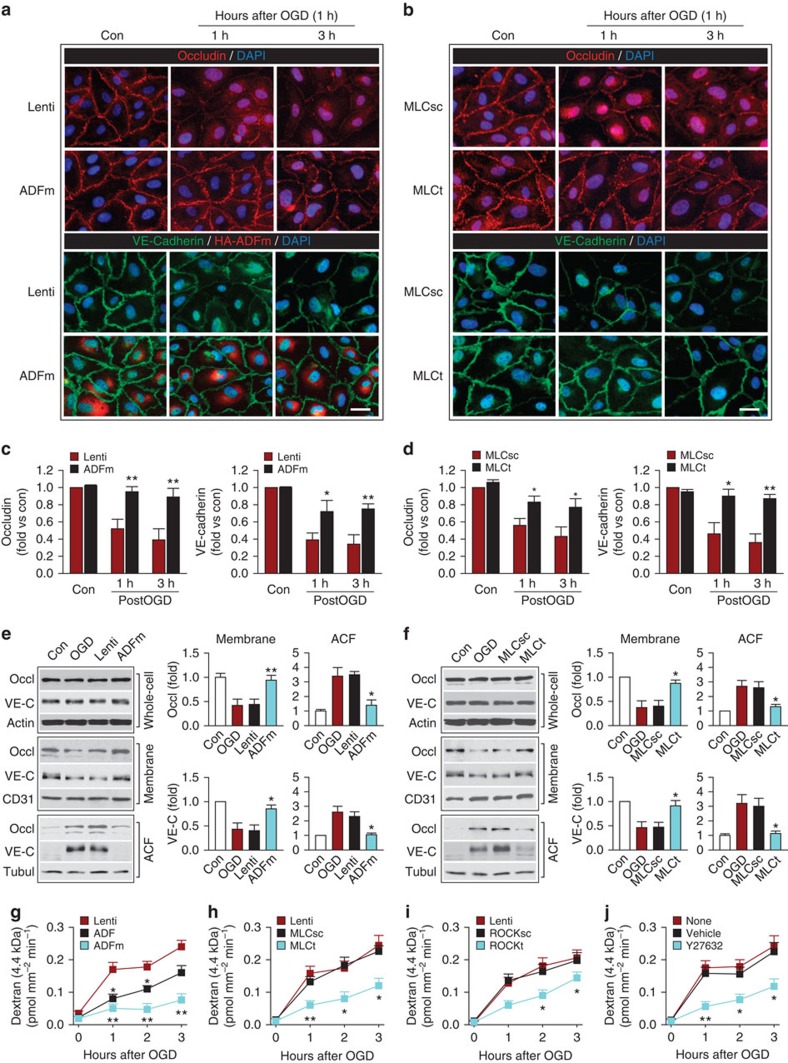
Cytoskeletal reorganization and JP redistribution in endothelial cells causes early paracellular hyperpermeability after oxygen–glucose deprivation *in vitro*. HBMECs were infected with Lenti, Lenti-ADF, Lenti-ADFm, Lenti-MLCsc, Lenti-MLCt, Lenti-ROCKsc or Lenti-ROCKt. In separate cultures, HBMECs were treated with vehicle or Y27632. Cells were then subjected to 1 h of OGD. (**a**,**b**) HBMECs were stained at 1 or 3 h after OGD for occludin (red) or VE-cadherin (green), and counterstained with DAPI (blue) for nuclear labelling. In ADFm-transfected cells, triple staining was performed for VE-cadherin (green), the HA tag (red) and DAPI nuclear labelling (blue), to show the cytosolic distribution of HA-ADFm. Scale bar, 30 μm. JPs are characteristically located at cell–cell contact sites under physiological, uninjured conditions. OGD resulted in a loss of occludin and VE-cadherin from the points of cell–cell contact, and this effect was inhibited by lentiviral ADFm overexpression or MLC knockdown. (**c**,**d**) The immunofluorescent staining intensity of plasma membrane occludin and VE-cadherin was quantified and expressed relative to non-OGD controls. Data represent four independent experiments. *P≤0.05, **P≤0.01 versus Lenti (**c**) or MLCsc (**d**). (**e**,**f**) Whole-cell extracts, the membrane fraction or the actin cytoskeleton fraction (ACF) were prepared at 1 h after OGD and immunoblotted for occludin (Occl), VE-cadherin (VE-C) and subfraction markers β-actin, CD31 or α-tubulin. Representative images and quantification of blots from the membrane fraction and ACF are presented. Data represent four independent experiments. *P≤0.05, **P≤0.01 versus Lenti (**e**) or MLCsc (**f**). (**g**–**j**) Lentivirus-infected or drug-treated HBMECs were cultured in the *in vitro* BBB model, and subjected to 1 h of OGD. The diffusion coefficient of the 4.4 kDa-dextran was measured at 0–3 h after OGD. Data represent four independent experiments. *P≤0.05, **P≤0.01 versus Lenti (**g**), MLCsc (**h**), ROCKsc (**i**) or vehicle (**j**).

Robust actin polymerization and stress fibre formation in ECs leads to cell contraction and redistribution/disassembly of JPs^16^. As expected, OGD weakened occludin and VE-cadherin expression at extracellular cell–cell contact sites and increased their expression in the cytosol and, perhaps, in the nuclear compartment as well (Fig. 4a–d), whereas ADFm overexpression or MLC knockdown abolished these changes. We further confirmed that the distribution of occludin and VE-cadherin was indeed shifted from the membrane fraction to the actin cytoskeleton fraction (ACF) in HBMECs at 1 h post OGD (Fig. 4e,f), while their total expression in the whole-cell lysates remained unchanged (Supplementary Fig. 3). The redistribution of occludin and VE-cadherin was likely linked to the tension generated from stress fibres and cell contraction, as it was blocked by ADFm overexpression or MLC knockdown.

Importantly, ADFm, and, to lesser extent, ADF overexpression, reduced EC permeability to both 4.4 kDa- (Fig. 4g) and 70 kDa-dextran (Supplementary Fig. 4a) at 1–6 h post OGD. MLC knockdown or ROCK knockdown/inhibition offered similar protection against barrier hyperpermeability (Fig. 4h–j; Supplementary Fig. 4b,c). The inhibitory effect of ADFm on EC permeability to the 4.4 kDa-dextran was also demonstrated using another *in vitro* BBB model composed of HBMEC and human astrocyte co-cultures^30^ (Supplementary Fig. 5). In summary, these data indicate that OGD-induced changes in EC structure, including actin polymerization and JP redistribution, occur at early stages (1–3 h) after I/R and contribute to barrier disruption. Blunting these alterations, either by overexpressing ADFm or inhibiting ROCK/MLC signalling, rescues the BBB from early hyperpermeability caused by OGD.

### ADFm blunts BBB damage and improves long-term function

**Figure 5:**
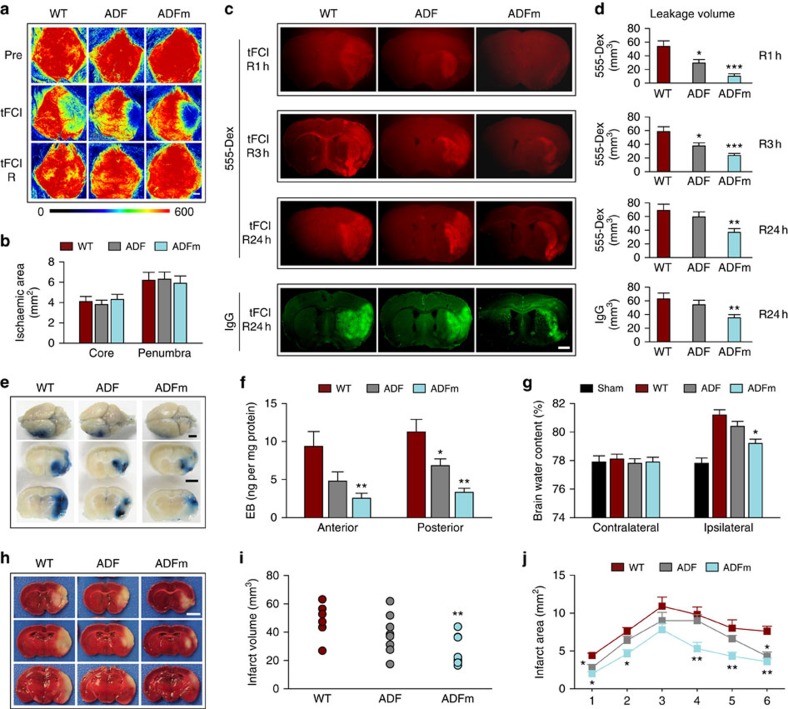
Transgenic overexpression of constitutively active ADF specifically within endothelial cells mitigates BBB disruption and reduces brain infarct size and brain oedema after focal cerebral ischaemia *in vivo*. tFCI was induced for 1 h in WT mice and in Tg mice with EC-specific overexpression of HA-tagged WT ADF or constitutively active mutant ADF (ADFm), as described in Methods. (**a**,**b**) Regional cerebral blood flow was monitored using two-dimensional laser speckle imaging techniques. (**a**) Representative images of CBF at 15 min before tFCI (Pre), 15 min after the onset of tFCI and at 15 min of reperfusion (R). Scale bar, 1 mm. (**b**) Ischaemic areas measured from laser speckle images were not affected by transgene expression in either the ischaemic core (0–20% residual CBF) or penumbra (20–30% residual CBF). *n*=6 mice per group. (**c**) Representative images showing the extravasation of Alexa555-dextran (3 kDa, red) or plasma IgG (green) into the brain parenchyma 1–24 h after tFCI. Scale bar, 1 mm. (**d**) Volume of leakage of Alexa555-dextran and endogenous IgG at indicated reperfusion time points. *n*=6 mice per group. (**e**) Extravasation of the Evans blue dye into the brain 24 h after tFCI in whole brains and thick coronal sections. Scale bar, 2 mm. (**f**) Evans blue content in brain tissue in anterior and posterior sections. *n*=6 mice per group. (**g**) Brain water content measured at 24 h after tFCI. *n*=6 mice per group. (**h**,**i**) Brain infarct volume at 48 h after tFCI was measured on TTC-stained coronal sections. Scale bar, 2 mm. *n*=6–8 mice per group. (**j**) Infarct areas at 48 h after tFCI, measured on six consecutive MAP2-stained sections (1 mm apart) spanning the MCA territory. *n*=6–8 mice per group. *P≤0.05, **P≤0.01, ***P≤0.001 versus WT.

To further elucidate the role of ADF in I/R-induced BBB damage, we generated conditional transgenic (Tg) mice with EC-specific overexpression of ADF or ADFm, by crossing loxP-flanked Tg-ADF^stop^ or Tg-ADFm^stop^ mice with Tek-cre mice^31^ in which the cre recombinase expression is restricted to ECs (Supplementary Fig. 6a–d). Expression of the transgene had no impact on cerebrovascular anatomy (Supplementary Fig. 6e,f; Supplementary Table 2). When tFCI was induced, transgene expression also did not alter physiological parameters (Supplementary Table 3) or hemodynamics (Fig. 5a,b; Supplementary Table 4). As expected, EC-targeted overexpression of ADFm markedly reduced the leakage of both the 3 kDa-dextran into ischaemic brain after 1, 3 and 24 h of reperfusion, and the leakage of plasma IgG at 24 h after ischaemia (Fig. 5c,d; Supplementary Fig. 7a–d). In contrast, EC-targeted overexpression of WT ADF significantly reduced the leakage of the 3 kDa-dextran at 1 and 3 h of reperfusion, but failed to reduce the leakage of either dextran or IgG at 24 h. The preservation of BBB integrity by ADFm was also confirmed by a reduction in Evans blue dye leakage and brain oedema at 24 h post tFCI (Fig. 5e–g; Supplementary Fig. 8). Notably, the ischaemic infarct was also significantly reduced in Tg-ADFm mice at 48 h after tFCI (Fig. 5h–j; Supplementary Fig. 9). It is likely that the degree of reduction of BBB leakage was not sufficiently reduced in WT ADF mice to elicit a similar effect on infarct volume as the constitutively active ADFm.

**Figure 6:**
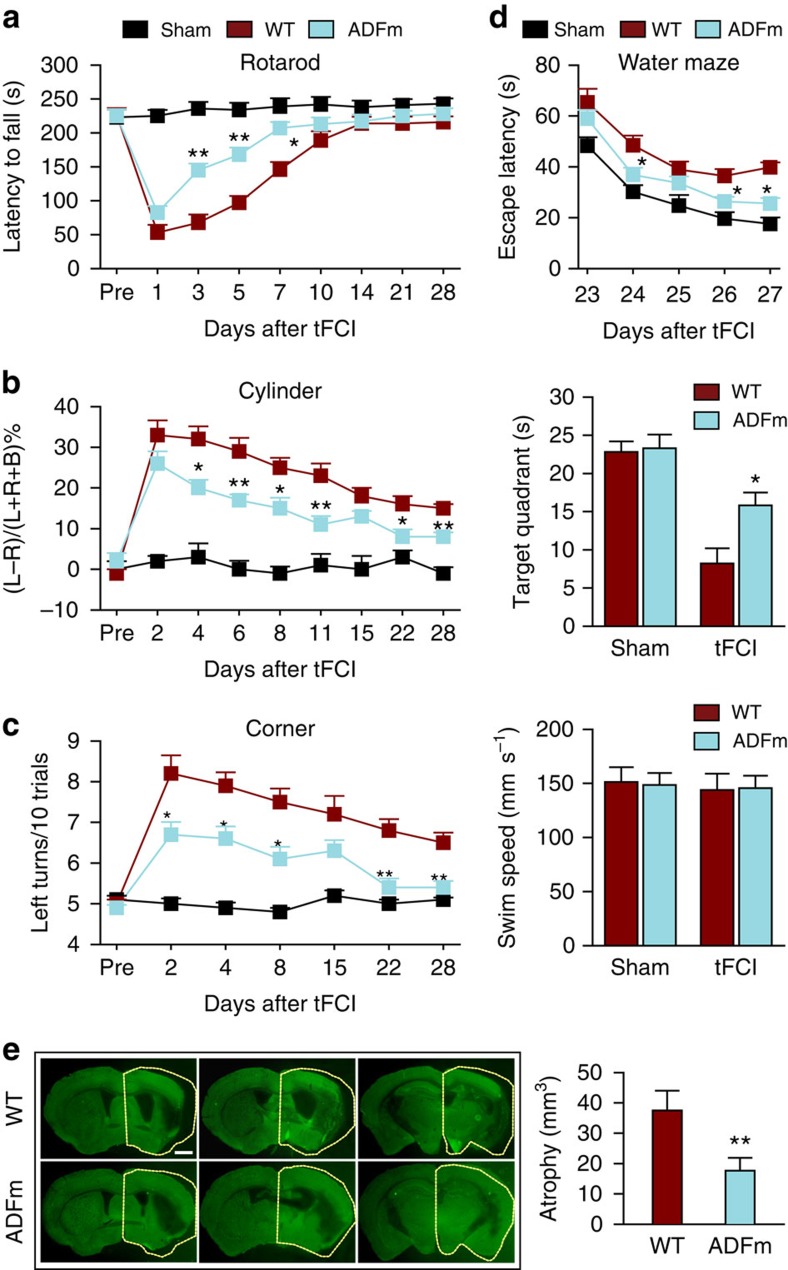
Targeted overexpression of ADFm specifically within endothelial cells improves long-term neurological functions after cerebral ischaemic injury *in vivo*. (**a**–**d**) Neurobehavioral tests were performed on WT and EC-specific ADFm-overexpressing mice before and 1–28 d after tFCI or sham operation. Sensorimotor deficits were evaluated by the rotarod test (**a**), cylinder test (**b**) and corner test (**c**). L, left; R, right; B, both forepaws in **b**. (**d**) Long-term cognitive deficits were assessed in the Morris water maze at 23–28 d after tFCI. The time needed for the animal to locate the submerged platform (escape latency) was measured from 23 to 27 d after tFCI. Spatial memory was evaluated at 28 d after tFCI by measuring the time spent in the target quadrant when the platform was removed. Gross locomotor functions, as reflected by similar swim speeds, were not affected by transgene expression. (**e**) Brain atrophy at 28 d after tFCI was quantified on MAP2 (green)-stained coronal sections. Dashed lines outline the relative area of the uninjured contralateral hemisphere to illustrate by comparison the area of ipsilateral atrophy. Scale bar, 1 mm. *n*=8–9 mice per group. *P≤0.05, **P≤0.01 versus WT. d, days.

To determine whether targeting early EC cytoskeletal changes provide sustained protection or simply delay injury, we evaluated long-term neurological functions after tFCI. Sensorimotor deficits and asymmetry were significantly alleviated up to 28 days after tFCI in Tg-ADFm mice compared with WT mice (Fig. 6a–c). The Morris water maze test revealed that long-term spatial cognitive functions were also improved by ADFm (Fig. 6d). Tg-ADFm animals also exhibited significant reductions in brain atrophy at 28 days after tFCI (Fig. 6e). Collectively, these data strongly suggest that ADFm prevents early (<3 h) BBB disruption and promotes long-term functional recovery after ischaemia by preserving the cytoskeletal microarchitecture of ECs.

### Actin polymerization elicits stress fibres after stroke

**Figure 7:**
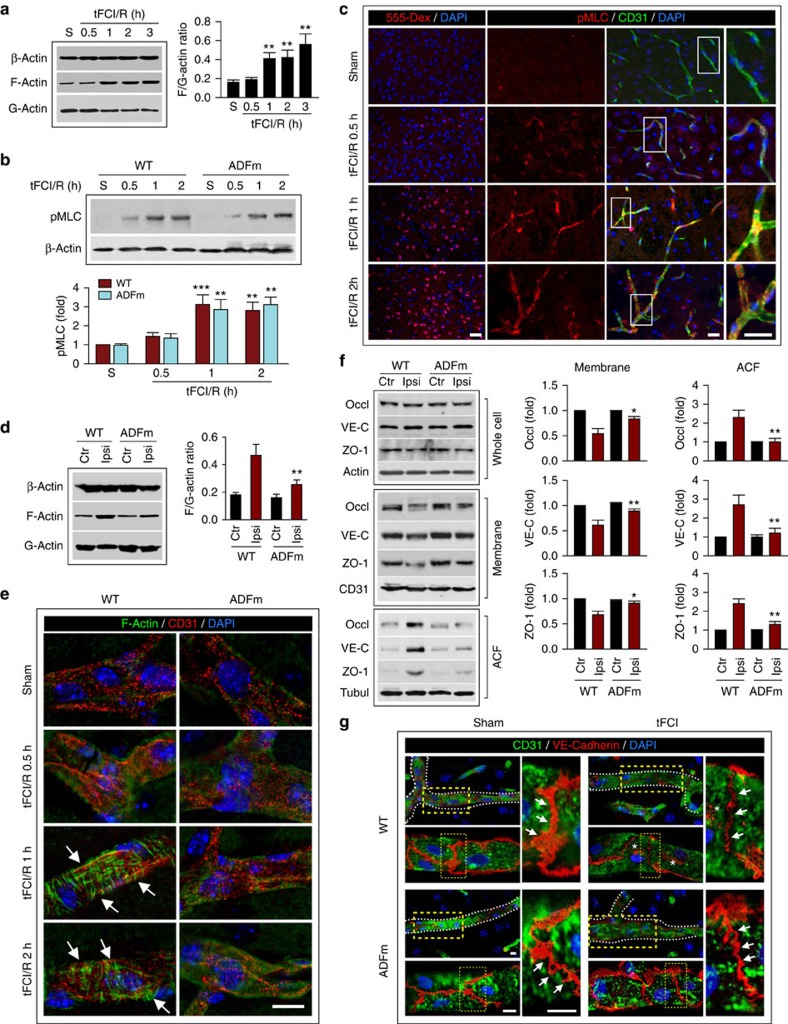
ADFm overexpression blunts early cytoskeletal alterations in ECs after tFCI. WT or Tg-ADFm mice received 1-h tFCI or sham (S) operation, and brain microvessel extracts were isolated at 0.5–3 h of reperfusion for western blotting analysis. The F/G-actin ratio (**a**) and pMLC (**b**) in ipsilateral cortical microvessels of WT mice were quantified. **P≤0.01, ***P≤0.001 versus sham. (**c**) Double-label immunostaining for pMLC and the endothelial marker CD31 in ipsilateral cortex. The leakage of the 3 kDa Alexa555-dextran is shown for each time point. Rectangle: the region enlarged in high-power images. Scale bar, 100 μm. (**d**) The F/G-actin ratio in microvessels at 1 h of reperfusion. **P≤0.01 versus WT. (**e**) Representative images showing the formation of F-actin^+^ stress fibres in CD31^+^ microvessels (arrow) in the ischaemic cortex. Scale bar, 10 μm. ADFm overexpression inhibited tFCI-induced formation of stress fibres. (**f**) Whole-cell extracts, the membrane fraction or the ACF were prepared from brain microvessel extracts at 1 h after tFCI and subsequently immunoblotted. Representative images and quantification of blots (normalized to WT contralateral) are presented. Endothelial ADFm overexpression suppressed tFCI-induced redistribution of JPs from the membrane fraction to the ACF. *n*=6 mice per group. *P≤0.05, **P≤0.01 versus WT. (**g**) Double-label immunostaining for VE-cadherin and CD31 in ipsilateral cortex at 1 h of reperfusion or after sham operation. Low-power images were shown (left top panel) with white lines delineating the shape of the vessels. Rectangles indicate regions where the high-power images were taken (shown in the left bottom panel). In non-ischaemic controls (both WT and Tg-ADFm brains), VE-cadherin immunofluorescence (arrows) was present predominantly at endothelial cell–cell contacts (cell membrane and extracellular space between cells; surrounding the cytosolic CD31 immunofluorescence). In WT brains, tFCI reduced the level of VE-cadherin immunofluorescence (arrows) in the cell–cell contacts of CD31^+^ endothelial cells but increased intracellular VE-cadherin immunofluorescence (asterisk) compared with non-ischaemic control brains. These changes were less frequently observed in Tg-ADFm brains. The enlarged images of VE-cadherin immunofluorescence (arrows) at endothelial cell–cell contacts (extracted from the rectangle regions at the left bottom panel) are shown on the right panel. Scale bar, 5 μm.

To determine whether the signalling events leading to JP changes *in vitro* also occur in brain vasculature *in vivo* after ischaemia, we isolated microvessels from the cerebral cortex at 0.5–2 or 3 h after tFCI and examined actin polymerization and phospho-MLC by western blots. In WT mice, actin polymerization and phospho-MLC were significantly increased at 1 h post tFCI and thereafter (Fig. 7a,b), consistent with the time course of BBB leakage of 3 kDa-dextran into the cortex (Fig. 7c). Triple-label immunofluorescence revealed that phospho-MLC was induced mainly in cerebral ECs (CD31^+^) at 1 and 2 h after tFCI (Fig. 7c).

Next, we determined the effect of ADFm on actin polymerization and MLC phosphorylation after ischaemia. Compared with WT littermates, EC-targeted Tg-ADFm mice showed significantly reduced levels of actin polymerization (Fig. 7d) but not MLC phosphorylation (Fig. 7b) in cerebral microvessels after tFCI. Consistent with the notion that stress fibre formation is the consequence of actin polymerization and/or MLC phosphorylation, triple-label immunofluorescence showed robust induction of stress fibres in ECs at 1 and 2 h after tFCI in WT, but not in ADFm Tg mice (Fig. 7e; Supplementary Fig. 10).

To test the hypothesis that actin polymerization and stress fibre formation lead to redistribution of JPs in ECs, we performed western blot analysis for occludin, VE-cadherin and ZO-1 using subcellular fractions from cortical microvessels after tFCI. Although total protein levels remained unchanged 1 h after tFCI, all three JPs redistributed from the membrane fraction to the ACF in microvessels of the ischaemic hemisphere (Fig. 7f; Supplementary Fig. 11). As expected, EC-targeted ADFm overexpression abolished JP redistribution. Furthermore, triple-label immunofluorescence confirmed the inhibitory effect of ADFm on ischaemia-induced redistribution of VE-cadherin in ECs (Fig. 7g).

### Early BBB damage enables degradation of JPs

**Figure 8:**
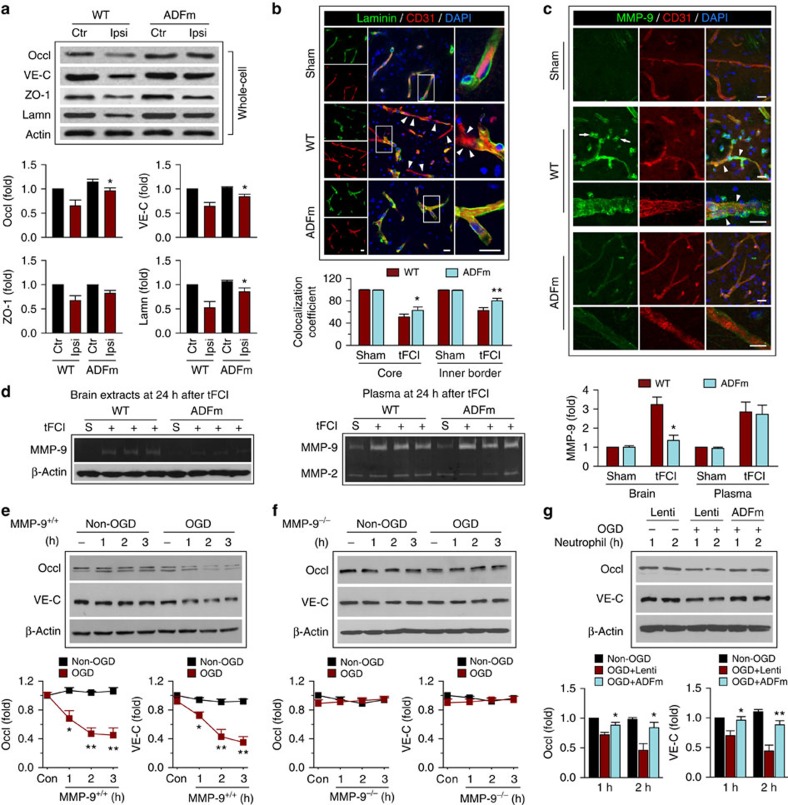
Early BBB damage leads to delayed JP degradation. (**a**–**d**) tFCI was induced in WT and Tg-ADFm mice for 1 h followed by 24 h of reperfusion. (**a**) Expression of JPs occludin, VE-cadherin and ZO-1, as well as the basal lamina protein laminin in whole-cell extracts from brain microvessels. Endothelial ADFm overexpression preserved occludin, VE-cadherin and laminin from tFCI-induced degradation. (**b**) Double-label immunostaining for laminin and CD31 in ipsilateral cortex. Consistent with its role as a basement membrane protein, laminin is distributed in the outer layer of CD31^+^ microvessels. Square: the region enlarged in high-power images. Arrowhead: partial loss of laminin protein (green signal). The overlap coefficient of laminin and CD31 immunofluorescence along the microvessels was calculated in the ischaemic core area and inner border zone, respectively. The coefficient was reduced after tFCI due to the partial loss of laminin, which was significantly attenuated in Tg-ADFm animals. (**c**) Double-label immunostaining for MMP-9 and CD31 in ipsilateral cortex. MMP-9 was upregulated after tFCI, mainly in CD31^+^ microvessels (arrowhead) and in infiltrated immune cells (arrow; see Supplementary Fig. 12a). ADFm overexpression abolished the upregulation of brain MMP-9. Scale bar, 50 μm. (**d**) MMP-9 or MMP-2 levels were measured by gelatin zymography in brain tissues and plasma. Endothelial ADFm overexpression blocked tFCI-induced MMP-9 elevation in the brain but not in the plasma. *n*=5–6 mice per group. *P≤0.05, **P≤0.01 versus WT. (**e**,**f**) Blood neutrophils were extracted from MMP-9^+/+^ or MMP-9^−/−^ mice 24 h after tFCI. HBMECs were exposed to 1 h of OGD, and immediately co-cultured with these neutrophils for 1–3 h. Expression of occludin and VE-cadherin in HBMECs was then examined. (**e**) OGD-challenged HBMECs became vulnerable to neutrophil-induced JP degradation. (**f**) The degradation of JPs was mediated by MMP-9, as MMP-9^−/−^ neutrophils failed to elicit protein degradation in OGD-challenged HBMECs. *P≤0.05, **P≤0.01 versus no neutrophil controls (Con). (**g**) HBMECs were infected with Lenti or Lenti-ADFm. After 48 h, cells were subjected to OGD, followed by co-culture with MMP-9^+/+^ neutrophils for 1–2 h. ADFm expression significantly preserved occludin and VE-cadherin against neutrophil MMP-9-mediated degradation. *P≤0.05, **P≤0.01 versus OGD+Lenti. Data represent four independent experiments.

Unexpectedly, when we extended the experimental time to 24 h post tFCI, Tg-ADFm animals displayed significant protection against tFCI-induced degradation of JPs (Fig. 8a; Supplementary Fig. 12). Loss of the basal lamina protein laminin was also significantly prevented (Fig. 8a,b). These results suggest that ADFm might influence brain MMP-9 levels at 24 h after tFCI, as MMP-9 is responsible for endothelial junction and basal lamina degradation at this time point^19^,^20^,^32^. As expected, MMP-9 expression was markedly elevated in the ipsilateral hemispheres at 24 h post tFCI, with predominant distribution in microvessels and infiltrated immune cells (Fig. 8c; Supplementary Fig. 13a). Gelatin zymography^33^ also revealed an elevation of both brain and plasma MMP-9 levels (Fig. 8d). EC-specific ADFm overexpression reduced MMP-9 expression in the ischaemic hemisphere. As the main sources of brain MMP-9 at this stage of I/R are infiltrating neutrophils and macrophages from the blood^32^, we also examined MMP-9 in plasma and blood neutrophils. Intriguingly, neither plasma nor neutrophil MMP-9 levels were altered in Tg mice (Fig. 8d; Supplementary Fig. 13b), supporting the hypothesis that ADFm specifically affects brain MMP-9 by modulating immune cell infiltration at the site of BBB injury.

### Early BBB rupture leads to immune cell infiltration

Our data thus far demonstrated that endothelial ADFm overexpression not only prevents EC junctional redistribution soon after tFCI but also attenuates subsequent MMP-9-mediated degradation of JPs and the ECM. Thus, we speculated that the early subtle structural changes in ECs may facilitate subsequent irreversible BBB damage. To test this hypothesis, we loaded activated neutrophils extracted from mouse blood at 24 h after tFCI onto HBMEC monolayers that had been subjected to OGD for 1 h or non-OGD conditions and then co-cultured for an additional 1–3 h. Incubation with activated neutrophils caused the degradation of JPs and induced prominent loss of occludin and VE-cadherin in HBMECs as early as 1 h after co-culture (Fig. 8e). This degradation was mediated by MMP-9 activity, as neutrophils extracted from MMP-9^−/−^ mice failed to elicit similar loss of JPs (Fig. 8f). Importantly, neutrophil-derived MMP-9 did not damage JPs in HBMECs under normal non-OGD conditions (Fig. 8e). These data suggest that OGD-challenged HBMECs become more vulnerable to neutrophil MMP-9-mediated degradation of JPs. Overexpression of ADFm prevented early junctional redistribution in ECs, and protected occludin and VE-cadherin from neutrophil MMP-9-mediated degradation (Fig. 8g). The OGD-weakened EC barrier also facilitated neutrophil transmigration across the HBMEC monolayer in the *in vitro* neutrophil migration model (Supplementary Fig. 14a). This migration of neutrophils was dependent on the CCL2–CCR2 system, as migration was significantly inhibited by the removal of CCL2 from the bottom chamber or in neutrophils from CCR2^−/−^ animals (Supplementary Fig. 14b). Neutrophil-derived MMP-9 appears to not only degrade BBB components but also enhance neutrophil infiltration as MMP-9^−/−^ neutrophils displayed markedly reduced migration (Supplementary Fig. 14c). Furthermore, ADFm overexpression or MLC knockdown in ECs blocked this neutrophil transmigration (Supplementary Fig. 14d).

**Figure 9:**
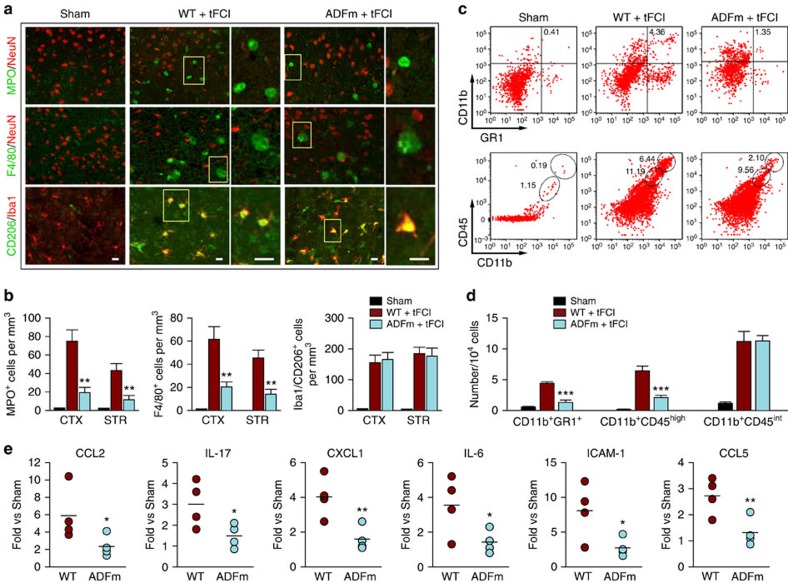
Overexpression of ADFm specifically in endothelial cells attenuates proinflammatory responses after ischaemic injury *in vivo*. tFCI was induced in WT or Tg-ADFm mice followed by 24 h of reperfusion. (**a**) Representative images from the inner border of infarction in the cortex after tFCI or the corresponding region after sham operation, showing immunostaining for the following markers: MPO (green, neutrophil), F4/80 (green, macrophage), NeuN (red, neuron), Iba1 (red, microglia/macrophage) and CD206 (green, activated M2 microglia/macrophage). Square: the region enlarged in high-power images. Scale bar, 20 μm. (**b**) MPO^+^, F4/80^+^ and Iba1/CD206^+^ cells were counted in the areas described in **a**, and data were expressed as the number of cells per mm^3^. *n*=6 mice per group. (**c**,**d**) Flow cytometric quantification of GR1^+^, CD45^high^ (circle) and CD45^int^ (oval) cells among the CD11b^+^ cell populations in WT and Tg-ADFm brains after tFCI or sham operation. Each fluorescence-activated cell sorting (FACS) sample was from two pooled brains. Data represent four independent experiments. Both immunofluorescent staining and FACS analyses demonstrated that endothelial ADFm overexpression impeded the infiltration of blood neutrophils/macrophages into the stroke brain without altering the numbers of resident microglia. (**e**) A panel of inflammatory markers was examined using the quantitative inflammation array in microvessel extracts from the ischaemic hemisphere at 24 h after tFCI. Levels of inflammatory markers were expressed relative to WT sham. ADFm overexpression significantly reduced the expression of several inflammation markers, including CCL2, IL-17, CXCL1, IL-6, ICAM-1 and CCL5. *n*=4 mice per group. *P≤0.05, **P≤0.01, ***P≤0.001 versus WT.

One possible explanation for the observation that ADFm reduced brain MMP-9 expression without changing the intrinsic production of MMP-9 in blood is that ADFm reduced the infiltration of MMP-9-producing blood cells (for example, neutrophils and macrophages). On the basis of our *in vitro* observation that ADFm blocked neutrophil transmigration, we further examined whether EC-targeted ADFm overexpression influences immune cell infiltration after tFCI. Following ischaemic brain injury, newly released chemoattractants form a steep concentration gradient at the interface of the ischaemic BBB to facilitate the infiltration of peripheral immune cells. Prominent parenchymal infiltration of MPO^+^ neutrophils and F4/80^+^ macrophages was already observed in the infarct and peri-infarct regions at 24 h after tFCI (Fig. 9a). ADFm expression in ECs blocked the infiltration of neutrophils and macrophages, but had little impact on the numbers of resident microglia (Fig. 9a,b). Similar results were obtained by fluorescence-activated cell sorting analyses (Fig. 9c,d). Both assays agreed that ADFm overexpression reduced the numbers of infiltrated CD11b^+^GR1^+^ cells (neutrophils and activated macrophages) and CD11b^+^CD45^high^ cells (macrophages) without parallel changes in the numbers of CD11b^+^CD45^int^ cells (resident microglia).

A panel of inflammatory markers was measured in brain microvessels to evaluate vascular neuroinflammation after ischaemic injury. Among the 40 inflammatory markers tested, 14 were increased at least 2.5-fold over sham controls (Fig. 9e; Supplementary Fig. 15). ADFm overexpression in ECs reduced the expression of 11 markers, including CCL2 and ICAM-1, which are known to facilitate macrophage and neutrophil infiltration after stroke. Taken together, these results demonstrate that ADFm overexpression reduces the vulnerability of the BBB to MMP-9-mediated damage and alleviates post-tFCI inflammatory responses by blocking early structural changes in ECs and BBB destruction. These effects may at least partially explain the prolonged protection of the BBB and of functional outcomes with ADFm overexpression.

## Discussion

**Figure 10:**
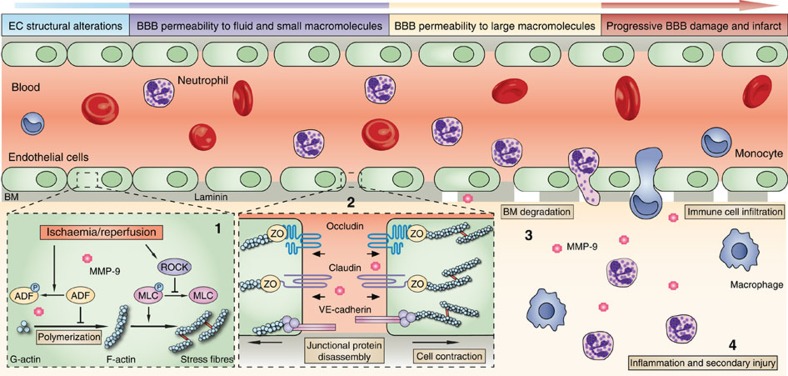
Proposed mechanisms underlying BBB disruption after stroke and the role of BBB leakage in the progression of permanent neurovascular damage. The evolution of BBB breakdown after cerebral ischaemia/reperfusion (I/R) progresses along the following steps: (1) I/R rapidly induces cytoskeletal alterations in brain microvascular endothelial cells (ECs). Actin polymerization is enhanced and F-actin^+^ stress fibres are formed inside injured ECs, resulting from both the activation of ROCK/MLC signalling and release from endogenous inhibition by ADF (supported by Figs 3 and 7). (2) Stress fibre formation causes EC contraction and disassembles tight junctional and adherens JPs (supported by Fig. 4) through junctional accessory proteins (for example, ZO-1). The disassembly and redistribution of JPs lead to subtle BBB hyperpermeability and induce extravasation of fluid and small macromolecules from blood into the central nervous system (supported by Fig. 1). (3) More importantly, the weakened barrier becomes more vulnerable to MMP-9-mediated degradation of JPs and basement membrane (BM) components (for example, laminin; supported by Fig. 8), further damaging the BBB and permitting eventual leakage of large macromolecules (supported by Fig. 2). (4) Peripheral immune cells, including neutrophils and macrophages, then transmigrate across the compromised BBB. Infiltrated immune cells release even more MMP-9 and other inflammatory mediators, degrading the ECM and causing irreversible BBB breakdown, secondary tissue injury and a sizeable infarct (supported by Fig. 9). By targeting the early structural changes in ECs, ADF overexpression shuts down the evolution of BBB disruption from the beginning. Continuous activity of ADF via overexpression of the nonphosphorylatable ADFm attenuates early BBB damage, as well as subsequent tissue injury, thereby offering long-term functional improvements (supported by Figs 5, 6, 7).

The present study characterizes the very early disruption of the BBB in experimental stroke, defines the underlying mechanism and investigates the role that this early disruption plays in the subsequent development of long-term neurovascular damage. As illustrated in Fig. 10, the major novel findings of the present study include the following: (1) soon after I/R (30–60 min), enhanced stress fibre formation and JP redistribution in brain microvascular ECs result in subtle impairment of BBB integrity and extravasation of small macromolecules (≤3 kDa) from blood into brain parenchyma. In contrast, gelatinase B/MMP-9 activity, the predominant mechanism previously thought to mediate post-stroke BBB disruption, contributes to leakage of larger macromolecules (⩾40 kDa) across the BBB in a relatively delayed manner (after more than 3 h). (2) The early, mild BBB breach increases vulnerability to subsequent MMP-9-mediated degradation and facilitates the infiltration of peripheral immune cells, leading to irreversible BBB breakdown and secondary expansion of tissue injury. (3) Targeting structural changes specifically within ECs prevents early BBB damage and blunts the progression of neuroinflammation, thereby eliciting long-term functional improvements after I/R.

The MMPs, and especially gelatinase B/MMP-9, have generally been held responsible for I/R-induced BBB disruption^2^, as multiple components of endothelial junctions and the basal lamina are well-known MMP-9 substrates and undergo proteolysis after ischaemia^19^. However, MMP inhibitors do not offer long-term protection of neurological function against ischaemia, probably due to the active participation of MMPs in tissue repair at chronic stages, such as the promotion of neurogenesis and angiogenesis^22^,^23^. The present study reveals that early structural alterations in ECs, including actin polymerization and the resulting JP redistribution, damage the BBB before the onset of junctional and ECM degradation by MMP-9. One limitation of the present study is that many intracellular proteins, including the actin cytoskeleton, are also substrates of MMP-9 (refs 34, 35). Although the current data do not rule out the possibility of actin cleavage by intracellular MMP-9 in ECs after ischaemia, this mechanism may not play an important role in the early BBB hyperpermeability, as the leakage of the 3 kDa-dextran still occurred in the used MMP-9^−/−^ mice (Fig. 1d,e).

The contributions of the early BBB breach to subsequent injury progression are twofold: first, the tension generated by stress fibres disassembles JPs, facilitating their internalization^36^. This disassembly may make the JPs and the ECM more accessible to MMP-9, as intact HBMECs were more resistant to neutrophil MMP-9-mediated junctional degradation than OGD-challenged HBMECs (Fig. 8). Second, widening of the EC junctions may promote the infiltration of circulating immune cells, which release MMP-9 to facilitate their transmigration and secrete even more inflammatory mediators, thereby contributing to secondary tissue injury. Targeting the very beginning of these pathological events, either by providing continuous ADF activity with ADFm or by inhibiting ROCK/MLC signalling, halts the progression of the injury, thereby conferring prolonged protection against BBB breakdown and neurological deficits. These long-term effects are notably distinct from the transient protective effects of inhibition of late-stage BBB breakdown in MMP-9 knockouts and suggest that it is more important to target the early breach of BBB integrity for halting the initiation of a toxic cascade. Thus, inhibition of MMP-9-mediated damage acts too late in the cascade of deleterious events to prevent the downstream consequences of the early BBB breach.

Under physiological conditions, the balance between actin polymerization and depolymerization is precisely regulated. In quiescent ECs, actin forms a cortical rim that is associated with cell–cell or cell–matrix adhesion complexes and serves as an anchor^37^. This anchoring is critical for normal EC function, such that disruption of the cortical actin rim causes hyperpermeability^38^. ECs experience continuous mechanical stress from hydrostatic pressure and fluid shear at the interface with blood^15^, which can stimulate the formation of stress fibres when excessive^39^. Excessive stress fibre formation disturbs the cortical actin rim and generates centripetal force throughout the cell^37^, which may break apart JPs and facilitate their internalization^36^. Our results demonstrate for the first time the importance of preserving the cortical actin cytoskeletal network shortly after I/R. The ADF/cofilin family plays a critical role in modulating actin dynamics and serves to inhibit actin polymerization and stress fibre formation^40^,^41^. The present study demonstrates that overexpression of nonphosphorylatable ADF provides continuous inhibition of actin polymerization and sustained protection of BBB integrity after ischaemia. These observations reveal EC cytoskeletal alterations as an important target for the preservation of BBB sealing efficiency. Following I/R, ADF/cofilin is quickly inactivated by phosphorylation, contributing to stress fibre formation and barrier disruption. The mechanism underlying ADF/cofilin inactivation by I/R is beyond the scope of the present study, although RhoA/ROCK and LIM kinases may play a role^29^,^42^.

Another mechanism that may contribute to early BBB dysfunction after stroke is trans-endothelial vesicular transport mediated by Caveolin1 (ref. 43). However, data obtained from both *in vivo* and *in vitro* systems suggest that Caveolin1-mediated transcellular mechanisms do not play a dominant role in BBB disruption up to 24 h after ischaemia (Supplementary Fig. 16). This discrepancy with previous work may be explained by differences in the severity of the ischaemic insults and the genetic background of the mice^43^. Further studies are warranted to investigate the participation of transcellular and paracellular pathways in BBB dysfunction.

Current understanding of neurovascular unit breakdown includes an appreciation of multicellular interactions and integrates the BBB with the neuroimmune interface^44^,^45^,^46^. I/R triggers the release of inflammatory mediators and proteases from activated ECs, and enhances leukocyte–EC interactions via selectins, adhesion molecules and chemokines/chemokine receptors^10^,^12^,^47^,^48^,^49^. For example, upregulation of endothelial ICAM-1 and CCL2 is important for neutrophil and monocytes/macrophage infiltration into the brain after stroke injury^47^. Adherence of leukocytes to the EC monolayer is followed by transmigration and extravasation, and further cytokine-mediated activation of ECs and astrocytes^10^. Leukocytes penetrate the endothelial basement membrane before subsequent penetration of the second parenchymal basement membrane and glia limitans in an MMP-2- and MMP-9-dependent manner^50^. Additional deleterious inflammatory mediators are then produced that promote further astrocyte secretion of chemokines to perpetuate the positive-feedback loop and elicit additional ECM degradation^32^,^51^,^52^,^53^,^54^,^55^. Within this established self-amplifying framework, the novelty of the present study lies in the demonstration of proposed MMP-9-independent BBB leakage shortly after acute stroke injury and a link between subtle, early hyperpermeability and subsequent, full-blown BBB degradation, parenchymal destruction and long-term neurological dysfunction.

One potential limitation of the present study is that the overexpression of ADF/ADFm was not restricted to the BBB, because the *Tek* promoter exists in virtually all ECs. As a result, we cannot rule out potential effects from peripheral ECs. On the other hand, our *in vitro* data confirm the contribution of brain ECs, thereby supporting the proposed mechanism.

Current stroke research is largely centred on neurons and brain parenchyma, whereas direct BBB protection has received far less attention. However, the failures of acute neuroprotective strategies in clinical trials suggest that future stroke research must expand its horizons to include the neurovascular unit. In addition, long-term responses to brain injury/repair should also be emphasized, to extend the temporal window for potential therapies^56^. For example, the use of tPA thrombolysis is severely limited due to the risk for haemorrhagic transformation when used beyond its therapeutic time window. Effective BBB-protective therapies therefore have the strong potential to improve the safety and efficacy of tPA treatment. The present study used a transient stroke model with intraluminal monofilament occlusion and lacks thrombolysis. Thus, it will be important to determine whether BBB-protective reagents are capable of reducing haemorrhages in models with tPA-induced reperfusion. Finally, age-related alterations in BBB functions, for example, loss/morphological changes in capillary ECs and a decrease in EC mitochondria^57^, may exacerbate ischaemic injury in the elderly and increase their risk for haemorrhagic transformation^58^,^59^. Further research to determine whether ADF can also improve neurological functions in aged animals is warranted.

In summary, the present study reveals a previously unexplored role for early BBB disruption in long-term outcomes after I/R injury. Inhibition of MMP-9 at late stages of ischaemic injury fails to lead to long-term neurological and histological protection. In contrast, prevention of early cytoskeletal changes in microvascular ECs attenuates BBB breakdown and secondary tissue injury, and ameliorates long-term neurological deficits. Our results have important implications outside the stroke field, in that early BBB damage appears to be a cause rather than only a result of parenchymal cell injury. Using endothelium-specific gene expression and analysing both early and long-term responses to injury, our studies are the first to demonstrate that subtle, early structural changes in the EC cytoskeleton are responsible for a self-amplifying cascade that culminates in robust inflammation and persistent loss of neurological function. Thus, our investigation of ADF as a tool to blunt EC-initiated inflammatory responses may lead to novel anti-inflammatory therapies. Furthermore, the unexpectedly early structural change in the EC cytoskeleton may be a novel target to help preserve BBB integrity in conditions including, but not limited to stroke.

## Methods

### General methodological information

Methodological details beyond the descriptions below are provided in Supplementary Methods.

### Animals

C57BL/6J, MMP-9^−/−^, CCR2^−/−^, Caveolin1^−/−^ and Tek-Cre mice were purchased from the Jackson Laboratory (Bar Harbor, Maine, USA). Tg-ADF^stop^ and Tg-ADFm^stop^ mice were generated as described in Supplementary Methods. Mice were housed in a temperature- and humidity-controlled animal facility with a 12-h light–dark cycle. Food and water were available *ad libitum*. All animal procedures were approved by the University of Pittsburgh Institutional Animal Care and Use Committee, and performed in accordance with the National Institutes of Health Guide for the Care and Use of Laboratory Animals. All efforts were made to minimize animal suffering and the number of animals used.

### tFCI ischaemia model

tFCI was induced in adult male mice (8–10 weeks old, 25–30 g) by intraluminal occlusion of the left middle cerebral artery (MCA) for 60 min^60^. Experimental procedures were performed following criteria derived from Stroke Therapy Academic Industry Roundtable group guidelines for preclinical evaluation of stroke therapeutics^61^. Briefly, mice were anaesthetized with 3% isoflurane in 67:30% N_2_O/O_2_ until they were unresponsive to the tail pinch test. Mice were then fitted with a nose cone blowing 1.5% isoflurane for anaesthesia maintenance. A 8-0 monofilament with silicon-coated tip was introduced into the common carotid artery, advanced to the origin of the MCA and left in place for 60 min. Rectal temperature was maintained at 37.0±0.5 °C during surgery through a temperature-controlled heating pad. Mean arterial blood pressure was monitored during surgery by a tail cuff, and arterial blood gas was analysed 15 min after the onset of ischaemia. Regional cerebral blood flow (CBF) was measured using laser Doppler flowmetry. Alternatively, cortical CBF was monitored using two-dimensional laser speckle techniques (Supplementary Methods). Animals that did not show a CBF reduction of at least 75% of baseline level or died after ischaemia induction (<10%) were excluded from further experimentation. Sham-operated animals underwent the same anaesthesia and surgical procedures, with the exception of MCA occlusion. For treatment with the MMP inhibitor GM6001, animals were randomly assigned to vehicle or GM6001 groups immediately after surgery. GM6001 was administrated through the tail vein (16 μg kg^−1^) at the onset of reperfusion. Treatments and all outcome assessments were performed by investigators blinded to experimental group assignments.

### Intravenous injection and detection of tracers

The fluorescent tracers Alexa Fluor 555-conjugated dextran (3 kDa, 200 μg per mouse; Invitrogen, Grand Island, NY, USA), FITC-conjugated dextran (2,000 kDa, 133 mg per mouse; Sigma-Aldrich, St Louis, Missouri, USA) and Alexa Fluor 488-conjugated bovine serum albumin (BSA; 60–70 kDa, 4.5 mg per mouse; Invitrogen) were used to assess BBB permeability after tFCI. To visualize the leakage of tracers through the impaired BBB, Alexa555-dextran and FITC-dextran were injected through the femoral vein 90 min before killing. Animals were deeply anaesthetized and transcardially perfused with 0.9% NaCl followed by 4% paraformaldehyde in PBS. Brains were collected and cryoprotected in 30% sucrose in PBS, and frozen serial coronal brain sections (30-μm thick) were prepared on a cryostat (CM1900, Leica, Bensheim, Germany). Sections were processed for direct fluorescent detection of Alexa555 and FITC, respectively. Images were acquired using an inverted Nikon Diaphot-300 fluorescence microscope equipped with a SPOT RT slider camera and Meta Series Software 5.0 (Molecular Devices, Sunnyvale, CA, USA). Six equally spaced sections encompassing the MCA territory were quantified for cross-sectional area of Alexa 555-dextran or FITC-dextran fluorescence. These areas were summed and multiplied by the distance between sections (1 mm) to yield a leakage volume in mm^3^. Alternatively, images were captured with an Olympus Fluoview FV1000 confocal microscope using FV10-ASW 2.0 software (Olympus America, Center Valley, PA, USA). The regions of interest (ROIs) from cortex and striatum were scanned at 512 × 512 pixels (233 × 233 μm^2^) with a × 40 objective lens. Images were analysed using NIH ImageJ software for the measurement of Alexa555 fluorescence intensity. Three sections were analysed for each brain and six ROIs were randomly selected from each section.

BBB integrity was assessed by measurement of the blood–brain transfer coefficient (*K*_i_) for Alexa555-dextran and Alexa488-BSA^62^. After intravenous injection of tracers, arterial blood was collected every 5 min for 20 min to measure the tracer concentration in plasma. Animals were then decapitated and hemispheres were weighed. Brains were homogenized in 50 mmol l^−1^ Tris buffer and centrifuged at 3,000 r.p.m. for 30 min. Methanol was added to the collected supernatant at a volume ratio of 1:1 and the mixture was centrifuged again under the same conditions. The fluorescent intensity of the supernatant, as well as of the blood samples was measured with a fluorescence reader at ex/em 544/590 nm or 485/540 nm for Alexa555-dextran or Alexa488-BSA, respectively. Concentrations of the tracers were calculated using a standard curve. Regional blood volume was determined in separate groups of mice decapitated 1 min after injection of tracers. The *K*_i_ was calculated as described previously^62^ according to the equation: *K*_i_=(*C*_br_−*V*_o_*C*_bl_)/∫*C*_pl_·d*t*, where *C*_br_ is the concentration of tracers in brain at decapitation (ng g^−1^), *C*_bl_ is the concentration of tracers in the final blood sample (ng ml^−1^), *V*_o_ is regional blood volume (ml g^−1^) and ∫*C*_pl_·d*t* is the integral of the arterial concentration of tracers over time *t*.

### Immunohistochemistry and image analysis

At multiple reperfusion time points after tFCI, mice were killed and coronal brain sections (30-μm thick) were prepared as described above. Alternatively, brains were processed for paraffin embedding and cutting (6-μm thick)^63^ to examine the distribution of VE-cadherin in ECs in microvessels. The sections were subsequently deparaffinized and prepared for staining as described previously^63^. Sections were blocked with 10% donkey serum in PBS for 1 h, followed by overnight incubation (4 °C) with the following primary antibodies: rabbit anti-MAP2 (1:200; Santa Cruz Biotechnology, Dallas, TX, USA), rabbit anti-NeuN (1:500; EMD Millipore, Billerica, MA, USA), goat anti-MMP-9 (1:200; R&D Systems, Minneapolis, MN, USA), rabbit anti-Iba1 (1:2,000; Wako, Richmond, VA, USA), rat anti-CD31 (1:200; BD Biosciences, San Jose, CA, USA), rat anti-F4/80 (Clone: BM8; 1:200; BioLegend, San Diego, CA, USA), rabbit anti-MPO (1:50; Abcam, Cambridge, MA, USA), rabbit polyclonal anti-VE-cadherin (1:200; Abcam, Cat.# ab33168), mouse anti-ZO-1 (1:100; Invitrogen), mouse anti-phospho-MLC (Ser19; 1:200; Cell Signaling), rabbit anti-laminin (1:200; Sigma-Aldrich), goat anti-CD206 (1:500; R&D Systems), rabbit anti-HA (1:1,000; Cell Signaling, Danvers, MA, USA). After washing, sections were incubated for 1 h at room temperature with donkey secondary antibodies conjugated with DyLight 488 or Cy3 (1:1,000, Jackson ImmunoResearch Laboratories, Inc., West Grove, PA, USA). F-actin staining was done using Alexa Flour 488-conjugated phalloidin (1:2,000; Invitrogen). Alternate sections from each experimental condition were incubated in all solutions except the primary antibodies to assess nonspecific staining. Sections were then counterstained with 4′,6-diamidino-2-phenylindole (Thermo Scientific, Pittsburgh, PA, USA) for 2 min at room temperature, mounted and coverslipped with Fluoromount-G (Southern Biotech, Birmingham, AL, USA). Fluorescence images were captured with confocal microscopy.

For measurements of endogenous IgG leakage, sections were blocked in 5% BSA for 1 h, followed by incubation with biotinylated horse anti-mouse IgG antibody (1:500; Vector Laboratories, Burlingame, CA, USA). Sections were then incubated with streptavidin/horseradish peroxidase reagents (Vectastain Elite ABC; Vector Laboratories) followed by 0.5 mg ml^−1^ diaminobenzidine with 0.05% H_2_O_2_. Images were acquired and IgG leakage volume was calculated on six sections that were equally spaced encompassing the MCA territory as described above. Alternatively, IgG immunofluorescence intensity was quantified on images obtained from confocal microscopy as described above.

Infarct volume or tissue atrophy was measured on seven equally spaced MAP2-stained sections encompassing the MCA territory using ImageJ. The actual infarct volumes with corrections for oedema were calculated as the volume of the contralateral hemisphere minus the non-infarcted volume of the ipsilateral hemisphere. Tissue atrophy was calculated as the volume of the contralateral hemisphere minus the ipsilateral hemisphere.

Cerebral neutrophil and macrophage infiltration, and microglial activation were quantified as we described previously^32^ by counting the numbers of MPO^+^ cells, F4/80^+^ cells and Iba1^+^/CD206^+^ cells, respectively. Cells were counted in both the cortex and striatum at two coronal levels (0.2 and −0.5 mm to bregma). All images were processed by a blinded observer with ImageJ for cell-based counting of automatically recognized cells. The mean was calculated from three fields in the cortex or striatum of each section and expressed as mean number of cells per square millimetre.

Co-localization of laminin and CD31 was determined by calculating the overlap coefficient of laminin and CD31 immunofluorescence signals, based on the method described previously^64^. Three sections were analysed for each brain, and six ROIs were randomly selected from the infarct core and inner infarct border, respectively, on each section.

### Brain water content measurements

Mice were killed with CO_2_ and brains were collected. Contralateral and ipsilateral hemispheres were separated and weighed, both while still wet and after oven drying at 100 °C for 48 h. Brain water content was calculated as: (wet weight−dry weight)/wet weight × 100%.

### Cerebral microvessel isolation

Microvessels were extracted from mouse brains as described previously^62^,^65^. Briefly, animals were decapitated and brains were collected and immersed in buffer A (103 mM NaCl, 4.7 mM KCl, 2.5 mM CaCl_2_, 1.2 mM KH_2_PO_4_, 1.2 mM MgSO_4_, 15 mM HEPES, pH 7.4) at 4 °C. Brains were then homogenized in fivefold volumes of buffer B (103 mM NaCl, 4.7 mM KCl, 2.5 mM CaCl_2_, 1.2 mM KH_2_PO_4_, 1.2 mM MgSO_4_, 15 mM HEPES, 25 mM HCO_3_, 10 mM glucose, 1 mM sodium pyruvate and 1 g per 100 ml dextran, pH 7.4) using a Teflon homogenizer. The homogenate was suspended in an equal volume of 25% BSA, and centrifuged at 5,800 *g* at 4 °C for 30 min. The pellet was resuspended in 10 ml buffer B and filtered through an 85-μm nylon mesh. The filtrate was passed over a 3 × 4-cm column containing 0.4-mm glass beads with a 44-μm nylon mesh at the bottom. Beads were washed with 400 ml of buffer B and only microvessels adhered to the glass beads in this step. Microvessels were recovered by gentle, repeated agitation of the glass beads in buffer B, and the supernatant was decanted and spun at 500 *g* for 5 min. The final pellet was processed for various biochemical assays as described below.

### Flow cytometry

Flow cytometric analysis was performed on single-cell suspensions from post-tFCI brains^32^. Briefly, brains were collected at 24 h after tFCI and flushed with PBS. Tissues were chopped into fine particles in complete RPMI 1640 medium supplemented with 10% fetal calf serum. Tissues were then incubated in 10 ml of digestion buffer (2% fetal calf serum, 1 mg ml^−1^ collagenase II, 0.5 mg ml^−1^ of DNase I in RPMI 1640 medium) for 1 h at 37 °C. The suspension was passed through a 70-μm cell strainer, resuspended in 40 ml of complete RPMI 1640 and centrifuged at 2,000 *g* for 10 min at 4 °C. Cells were fractionated on a 30–60% percoll gradient (GE Healthcare BioSciences, Pittsburgh, PA, USA) at 1,000 *g* for 25 min. The mononuclear cells at the interface were washed before staining. Isolated cells were resuspended at 1 × 10^6^ ml^−1^ and stained with anti-mouse CD11b, CD45, GR1 and the appropriate isotype controls following the manufacturer's instructions (eBioscience, San Diego, CA, USA). Flow cytometric analysis was performed using a FACS flow cytometer (BD Biosciences).

### Quantitative inflammation array

Brain microvessels were isolated from the ipsilateral hemisphere at 24 h after tFCI or from the corresponding hemisphere of sham-operated animals, as described above. The concentration of 40 cytokines in microvessel extracts was determined using the Quantibody Mouse Inflammation Array Kit according to manufacturer's instructions (RayBiotech, Inc., Norcross, GA, USA). The concentrations of various cytokines were expressed relative to sham brains.

### *In vitro* BBB model

Primary HBMECs were purchased from Cell Systems (ACBRI 376, Kirkland, WA, USA). HBMECs were grown in Clonetics EGM-2 MV media (CC-3202, Lonza, Walkersville, MD, USA), and only up to eight passages were used for experiments. The *in vitro* BBB model was established in cell culture inserts as described before^66^. The transwell PET membranes (0.4-μm pore, 11-mm diameter; Corning, Lowell, MA, USA) were coated with collagen (15 μg ml^−1^) and fibronectin (30 μg ml^−1^). HBMECs were seeded onto the membrane at a density of 2.5 × 10^5^ cells per membrane (Fig. 2a). Cultures were maintained at 37 °C in humidified 95% air and 5% CO_2_ for 4 days to reach confluence. To assess paracellular permeability, 4.4 kDa TRITC-dextran or 70 kDa FITC-dextran (Sigma-Aldrich) were added into the luminal chamber at a concentration of 2 mg ml^−1^ in 500 μl media. Fluorescence intensity was measured with a fluorescence reader at 30-min intervals for 1–6 h by removing 30 μl media from the lower (abluminal) chamber. The concentrations of tracers in samples were calculated from a standard curve fitted using known concentrations of tracers. Thirty microlitre fresh media was added after each reading. Paracellular permeability was calculated by measuring the diffusion coefficient of tracers from the luminal to the abluminal chamber^11^.

### Oxygen–glucose deprivation

To model ischaemia *in vitro*, cultured HBMECs were exposed to transient OGD for 60 min (ref. 60). Briefly, culture medium was replaced with glucose-free medium, and cultures were placed in a Billups-Rothenberg modular incubator chamber (Del Mar, San Diego, CA, USA), which was flushed for 5 min with 95% argon and 5% CO_2_, and then sealed. The chamber was placed in a water-jacketed incubator (Forma, Thermo Fisher Scientific, Waltham, MA, USA) at 37 °C for 60 min and then returned to 95% air, 5% CO_2_ and glucose-containing medium for a period of time indicated in each experiment. Control glucose-containing cultures were incubated for the same period of time at 37 °C in humidified 95% air and 5% CO_2_. The MMP inhibitor GM6001 (1 or 3 μM) or the ROCK inhibitor Y27632 (10 μM) was added 30 min before OGD and remained in the media during and after OGD.

### Immunocytochemistry

HBMECs grown on collagen-coated coverslips in 24-well culture plates were fixed with 4% paraformaldehyde followed by blocking with donkey serum in 0.3 M glycine in PBS for 1 h at room temperature. The cells were then incubated with the following primary antibodies overnight at 4 °C: rabbit anti-VE-cadherin (1:200; Abcam), rabbit anti-occludin (1:200; Invitrogen) and rabbit anti-HA (1:500; Abcam). After rinses in PBS, cells were incubated with donkey secondary antibodies conjugated with DyLight 488 or Cy3 (1:1,000, Jackson ImmunoResearch Laboratories, Inc.). F-actin staining was done using Alexa Flour 488-conjugated phalloidin (1:2,000; Invitrogen). Cells were then counterstained with 4′,6-diamidino-2-phenylindole for nuclear labelling. After PBS rinses, coverslips were mounted on glass slides with antifade Vectashield solution (Vector Laboratories). Fluorescence images were captured with a confocal microscope. Immunofluorescence intensity of extracellular occludin and VE-cadherin was quantified by ImageJ.

### Preparation of subcellular fractions

To examine the distribution of TJ and AJ proteins in OGD-treated HBMECs or post-ischaemic brain microvessels, the membrane fraction and ACF were extracted using the ProteoExtract Subcellular Proteome Extraction Kit (Calbiochem, Billerica, MA, USA) according to the manufacturer's instructions. Fractions were then processed for western blotting as described below.

### Neutrophil and HBMEC co-cultures

Primary mouse neutrophils from blood were isolated using the EasySep Mouse Neutrophil Enrichment Kit (STEMCELL Technologies, Vancouver, Canada) according to the manufacturer's instructions and as we described previously^32^. Cultured HBMECs were subjected to 1-h OGD or control non-OGD conditions. Isolated neutrophils were placed on top of the HBMEC monolayer immediately after pre-exposure to OGD or non-OGD, at a density of 2 × 10^5^ cells per ml for HBMECs and 5 × 10^5^ cells per ml for neutrophils. The HBMEC–neutrophil co-cultures were maintained at 37 °C for 1–3 h and neutrophils were washed out. HBMECs were collected and processed for western blotting analyses as described below.

### Western blotting

Protein isolation from brain tissues or cultured HBMECs was performed as we described previously^67^. Western blots were performed using the standard SDS–polyacrylamide gel electrophoresis method and enhanced chemiluminescence detection reagents (GE Healthcare Biosciences). Immunoreactivity was semi-quantitatively measured by gel densitometric scanning and analysed with the MCID image analysis system (Imaging Research, Inc.). Images of blots were cropped for presentation, and full-size images are presented in Supplementary Fig. 17. Antibodies against the following proteins were used: rabbit anti-occludin (1:1,000; Invitrogen), rabbit anti-claudin-5 (1:500; EMD Millipore), rabbit anti-VE-cadherin (1:1,000; Abcam), rabbit anti-ZO-1 (1:250; Abcam), rabbit anti-phospho-ADF/cofilin (Ser3; 1:1,000; Cell Signaling), rabbit anti-total-ADF/cofilin (1:1,000; Cell Signaling), rabbit anti-ROCK1/2 (1:1,000; Cell Signaling), mouse anti-phospho-MLC (Ser19; 1:1,000; Cell Signaling), rabbit anti-CD31 (1:1,000; Abcam), rabbit anti-laminin (1:1,000; Sigma-Aldrich), rabbit anti-Caveolin1 (1:1,000; Cell Signalling), rabbit anti-α-tubulin (1:1,000; Abcam) and mouse anti-β-actin antibody (1:2,000; Sigma-Aldrich).

### Statistical analyses

Data are presented as mean±s.e.m. Comparison of means between two groups was accomplished by the Student's *t*-test (two tailed). Differences in means among multiple groups were analysed using one- or two-way analysis of variance followed by the Bonferroni/Dunn *post hoc* correction. A *P* value <0.05 was considered statistically significant.

## Additional information

**How to cite this article:** Shi, Y. *et al*. Rapid endothelial cytoskeletal reorganization enables early blood–brain barrier disruption and long-term ischaemic reperfusion brain injury. *Nat. Commun.* 7:10523 doi: 10.1038/ncomms10523 (2016).

## Supplementary Material

Supplementary Information Supplementary Figures 1-17, Supplementary Tables 1-4, Supplementary Methods and Supplementary References.
