# Publisher Correction: Rapid endothelial cytoskeletal reorganization enables early blood–brain barrier disruption and long-term ischaemic reperfusion brain injury

**DOI:** 10.1038/s41467-020-18263-5

**Published:** 2020-08-25

**Authors:** Yejie Shi, Lili Zhang, Hongjian Pu, Leilei Mao, Xiaoming Hu, Xiaoyan Jiang, Na Xu, R. Anne Stetler, Feng Zhang, Xiangrong Liu, Rehana K. Leak, Richard F. Keep, Xunming Ji, Jun Chen

**Affiliations:** 1 Center of Cerebrovascular Disease Research, University of Pittsburgh School of Medicine, Pittsburgh, PA 15213 USA; 2 Geriatric Research, Educational and Clinical Center, Veterans Affairs Pittsburgh Health Care System, Pittsburgh, PA 15261 USA; 3 State Key Laboratory of Medical Neurobiology, Institute of Brain Science and the Collaborative Innovation Center for Brain Science, Fudan University, Shanghai, 200032 China; 4 China-America Institute of Neuroscience, Xuanwu Hospital, Capital Medical University, Beijing, 100053 China; 5 Division of Pharmaceutical Sciences, Mylan School of Pharmacy, Duquesne University, Pittsburgh, PA 15282 USA; 6 Department of Neurosurgery, University of Michigan, Ann Arbor, MI 48109 USA

Correction to: *Nature Communications* 10.1038/ncomms10523, published online 27 January 2016.

The original version of this Article contained an error in author affiliations. Yejie Shi was incorrectly associated with China-America Institute of Neuroscience, Xuanwu Hospital, Capital Medical University, Beijing 100053, China, instead of Geriatric Research, Educational and Clinical Center, Veterans Affairs Pittsburgh Health Care System, Pittsburgh, PA 15261, USA. This has not been corrected in both the PDF and HTML versions of the Article.

