# Supplementary material for: Rapid endothelial cytoskeletal reorganization enables early blood–brain barrier disruption and long-term ischaemic reperfusion brain injury
Source: Nat Commun. 2016 Jan 27;7:10523. doi: 10.1038/ncomms10523 (PMC4737895; doi:10.1038/ncomms10523)
Supplement: Supplementary Information — Supplementary Figures 1-17, Supplementary Tables 1-4, Supplementary Methods and Supplementary References. [file ncomms10523-s1.pdf]

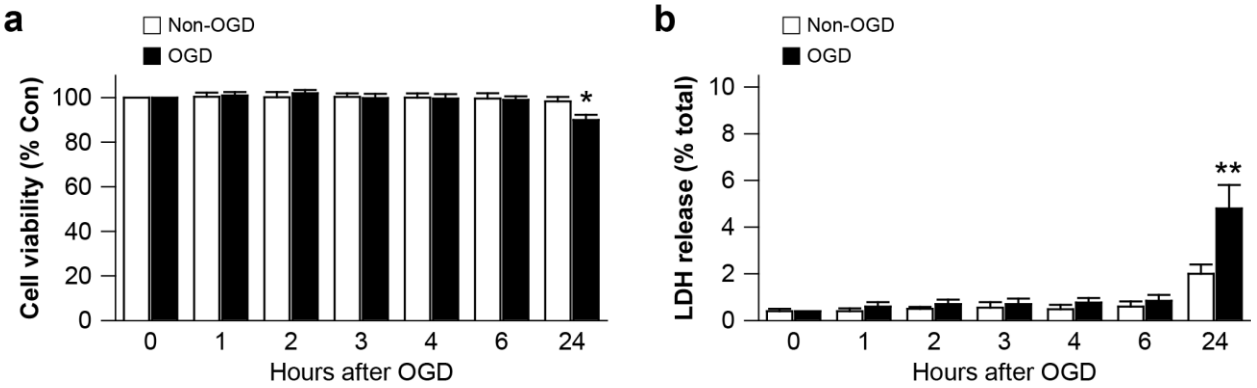

Supplementary Figure 1 | OGD causes overt cell death in cultured HBMECs after 24 h.

HBMECs were exposed to 1 h of OGD or control non-OGD conditions. (**a**) Cell viability was assessed using the 3-(4,5-dimethylthiazol-2-yl)-2,5-diphenyltetrazolium bromide (MTT) assay 0-24 h after OGD. Data were expressed as percentage of non-OGD control at 0 h. (**b**) Lactate dehydrogenase (LDH) release from HBMECs was measured 0-24 h after OGD and expressed as percentage of the maximum LDH activity of total lysed cells. Data represent 5 independent experiments. *$p \leq 0.05$, **$p \leq 0.01$ *versus* non-OGD.

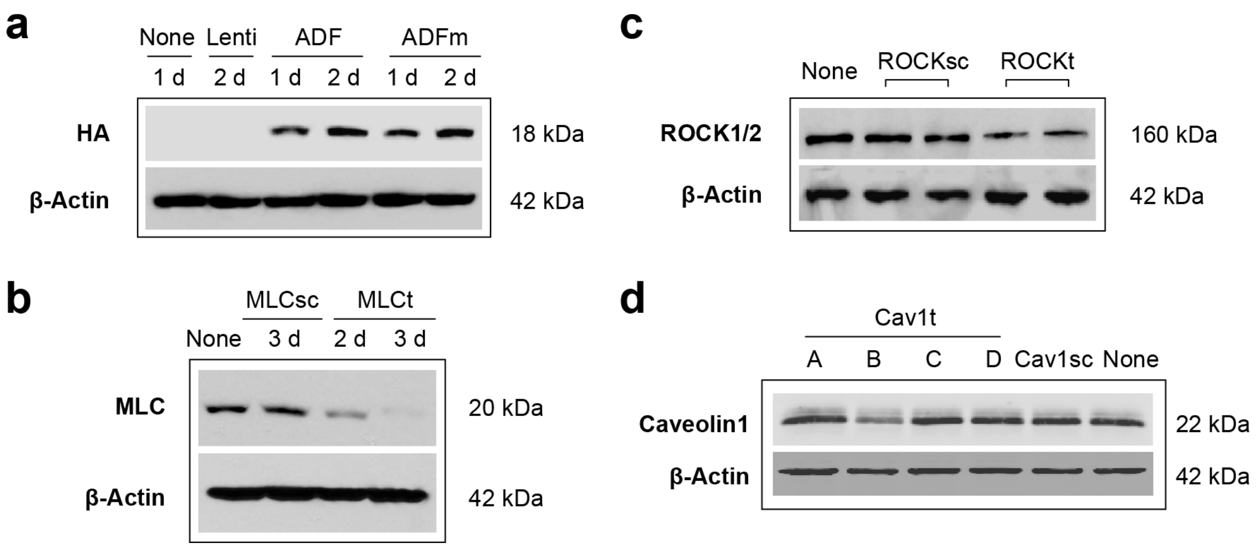

Supplementary Figure 2 | Lentivirus-mediated gene expression or knockdown in cultured HBMECs.

HBMECs were infected with control empty lentivirus (Lenti), or lentiviral vectors carrying HA-tagged wild-type ADF (ADF), HA-tagged constitutively active mutant ADF (ADFm), MLC-targeting shRNA (MLCt), ROCK-targeting shRNA (ROCKt), Caveolin1-targeting shRNA (Cav1t, four different sequences A-D), or non-targeting scrambled sequences (MLCsc, ROCKsc, or Cav1sc). (**a**) ADF or ADFm transduction in HBMECs was confirmed by Western blotting for the HA tag 1 and 2 d after infection. (**b**) MLC expression was significantly reduced 2 and 3 d after lentiviral knockdown. (**c**) ROCK1/2 expression was reduced 3 d after infection. (**d**) Caveolin1 expression was reduced by shRNA with Sequence B 4 d after infection. In the subsequent experiments (see Supplementary Fig. 16), Sequence B was used to knockdown Caveolin1. In all blots, β-actin was used as an internal loading control.

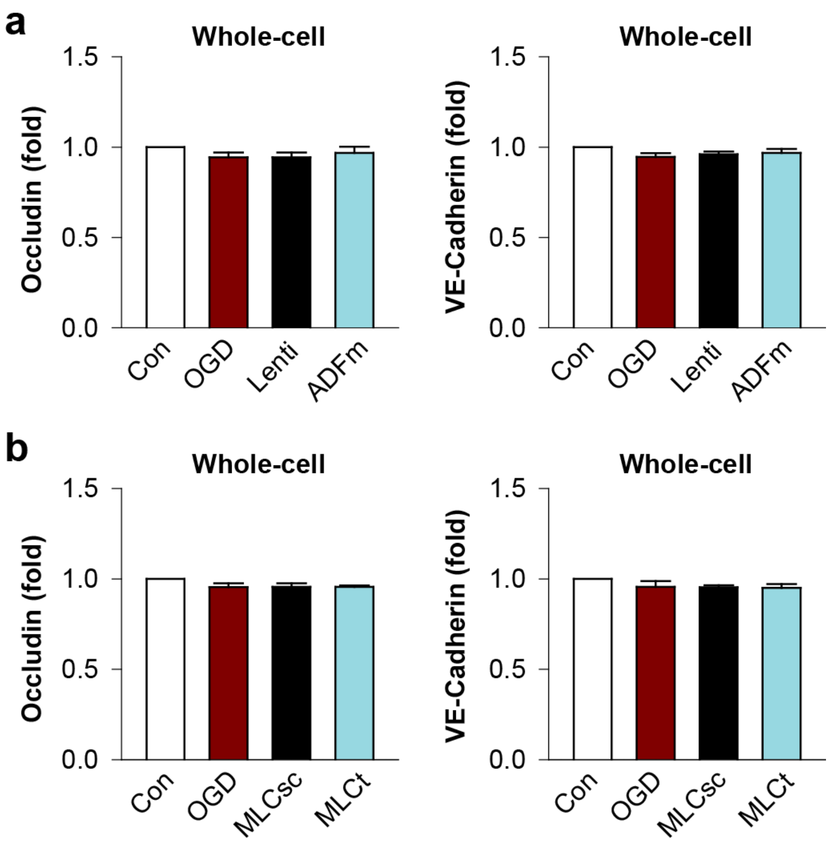

Supplementary Figure 3 | Overexpression of ADFm or knockdown of MLC does not alter the expression of junctional proteins in whole-cell lysates from HBMECs.

(**a**) HBMECs were infected with control empty lentivirus (Lenti), or lentiviral vectors carrying HA-tagged constitutively active mutant ADF (ADFm) for 48 h. (**b**) HBMECs were infected with lentiviral vectors carrying MLC-targeting shRNA (MLCt), or non-targeting scrambled sequences (MLCsc) for 72 h. Transfected or non-transfected cells were subjected to 1 h of OGD. Expression of occludin and VE-cadherin was quantified on Western blots from whole cell extracts 1 h after completion of OGD (see Fig. 4e,f), and expressed relative to non-OGD controls (Con). Data represent 4 independent experiments.

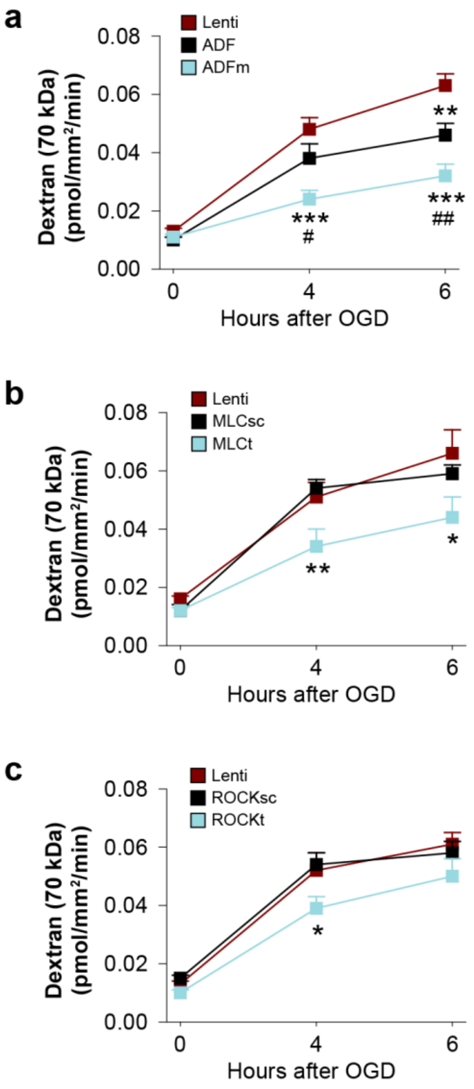

Supplementary Figure 4 | Overexpression of ADFm in HBMECs reduces barrier permeability to 70 kDa FITC-dextran after OGD.

Monolayers of HBMECs were infected with control empty lentivirus (Lenti), or lentiviral vectors carrying HA-tagged wild-type ADF (ADF), HA-tagged constitutively active mutant ADF (ADFm), MLC-targeting shRNA (MLCt), ROCK-targeting shRNA (ROCKt), or non-targeting scrambled sequences (MLCsc and ROCKsc). After 48 h of gene overexpression (ADF or ADFm) or after 72 h of gene knockdown (MLC or ROCK), HBMECs grown to confluence were subjected to 1 h of OGD. The diffusion coefficient of the 70 kDa FITC-dextran across the HBMEC monolayer was measured at 0, 4 and 6 h after OGD. Data represent 4 independent experiments. *$p \leq 0.05$, **$p \leq 0.01$, ***$p \leq 0.001$ *versus* Lenti (**a**), MLCsc (**b**), or ROCKsc (**c**). $^{\#}p \leq 0.05$, $^{\#\#}p \leq 0.01$ *versus* ADF.

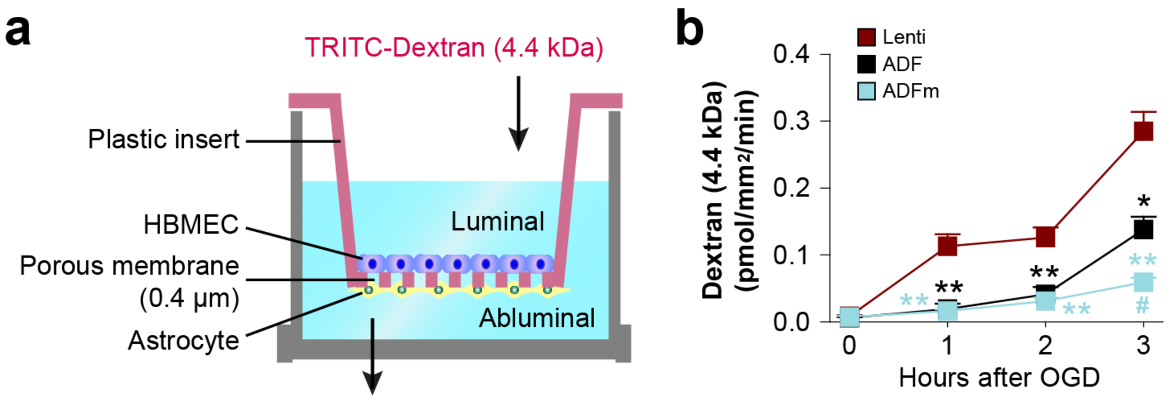

Supplementary Figure 5 | ADFm overexpression in HBMECs reduces barrier permeability after OGD in an EC-astrocyte co-culture model.

(**a**) Illustration of the *in vitro* BBB model consisting of HBMECs and human astrocytes. Monolayers of HBMECs were infected with control empty lentivirus (Lenti), or lentiviral vectors carrying HA-tagged ADF or ADFm. After 48 h of gene overexpression, HBMECs were cultured with astrocytes, separated by a membrane in the cell culture insert, and were subjected to 1 h of OGD. Paracellular permeability was determined by measuring the luminal to abluminal diffusion coefficient of a 4.4 kDa TRITC-dextran. (**b**) The diffusion coefficient of the 4.4 kDa dextran was measured 0-3 h after OGD. Data represent 4 independent experiments. *$p \le 0.05$, **$p \le 0.01$ *versus* Lenti. $^{\#}p \le 0.05$ *versus* ADF.

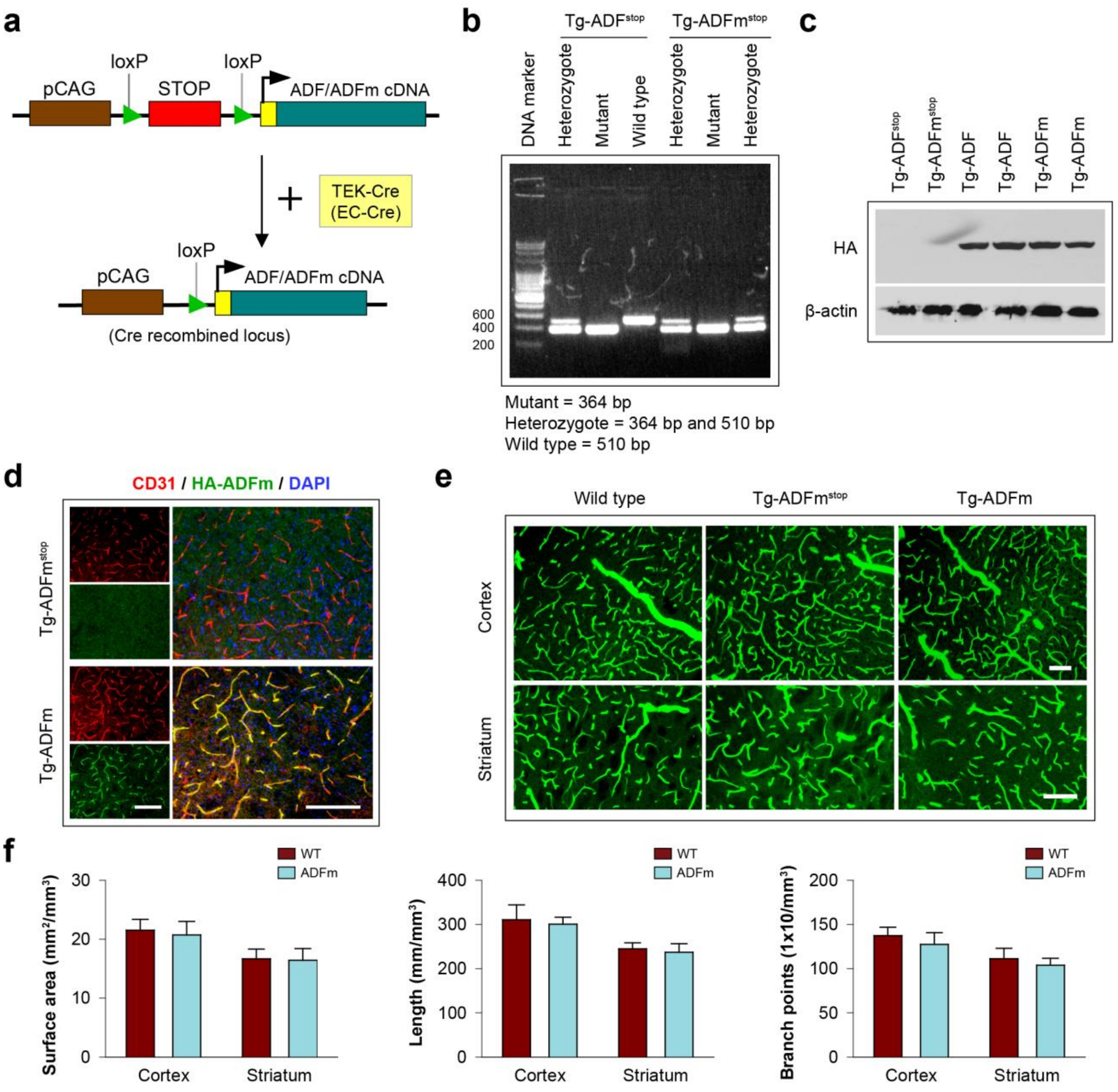

Supplementary Figure 6 | Generation and characterization of EC-targeted ADF- or ADFm-overexpressing Tg mice.

(**a**) Illustration of the targeting strategy used for generating EC-specific ADF- or ADFm-overexpressing mice. cDNA of full-length WT human ADF or its constitutively active mutant (ADFm) bearing single amino acid substitution (S3A) was targeted to the *ROSA26* locus downstream of a stop codon flanked by two loxP sites, driven by the CAG promoter (Tg-ADF^{stop} or Tg-ADFm^{stop}). Tg-ADF^{stop} or Tg-ADFm^{stop} mice were crossed with Tek-Cre mice, in which the Cre recombinase expression is driven by the *Tek* (endothelial-specific receptor tyrosine kinase) promoter and thus restricted to ECs.

In the presence of Cre recombinase, the stop codon is excised and a 5' HA-tagged ADF or ADFm protein is expressed specifically in ECs (Tg-ADF or Tg-ADFm). (**b**) A typical genotyping gel showing analysis of PCR products with agarose gel electrophoresis. The predicted size of the WT or mutant allele is 510 bp and 364 bp, respectively. (**c**) Overexpression of ADF or ADFm in ECs was confirmed by Western blotting for the HA tag in brain protein extracts. HA was expressed in Tg-ADF and Tg-ADFm animals but not in uncrossed Tg-ADF^{stop} or Tg-ADFm^{stop} animals. (**d**) Double-label immunostaining for the endothelial marker CD31 (*red*) and the HA tag (*green*) in the cerebral cortex of uncrossed Tg-ADFm^{stop} or crossed Tg-ADFm mice. ADFm was not expressed in uncrossed Tg-ADFm^{stop} brains. In Tg-ADFm brains, expression of ADFm was predominantly in microvessels, as shown by colocalization of HA and CD31 in *yellow*. *Scale bar*: 500 µm. (**e**) Cerebral microvasculature was examined in the cortex and striatum of WT, Tg-ADFm^{stop}, and Tg-ADFm brains by perfusion with FITC-lectin (*green*). *Scale bar*: 200 µm. (**f**) The vascular surface area, length, and branch points were quantified on lectin-labeled images. n=6 mice per group. No significant difference was observed in microvessel distribution and anatomy between WT and Tg animals.

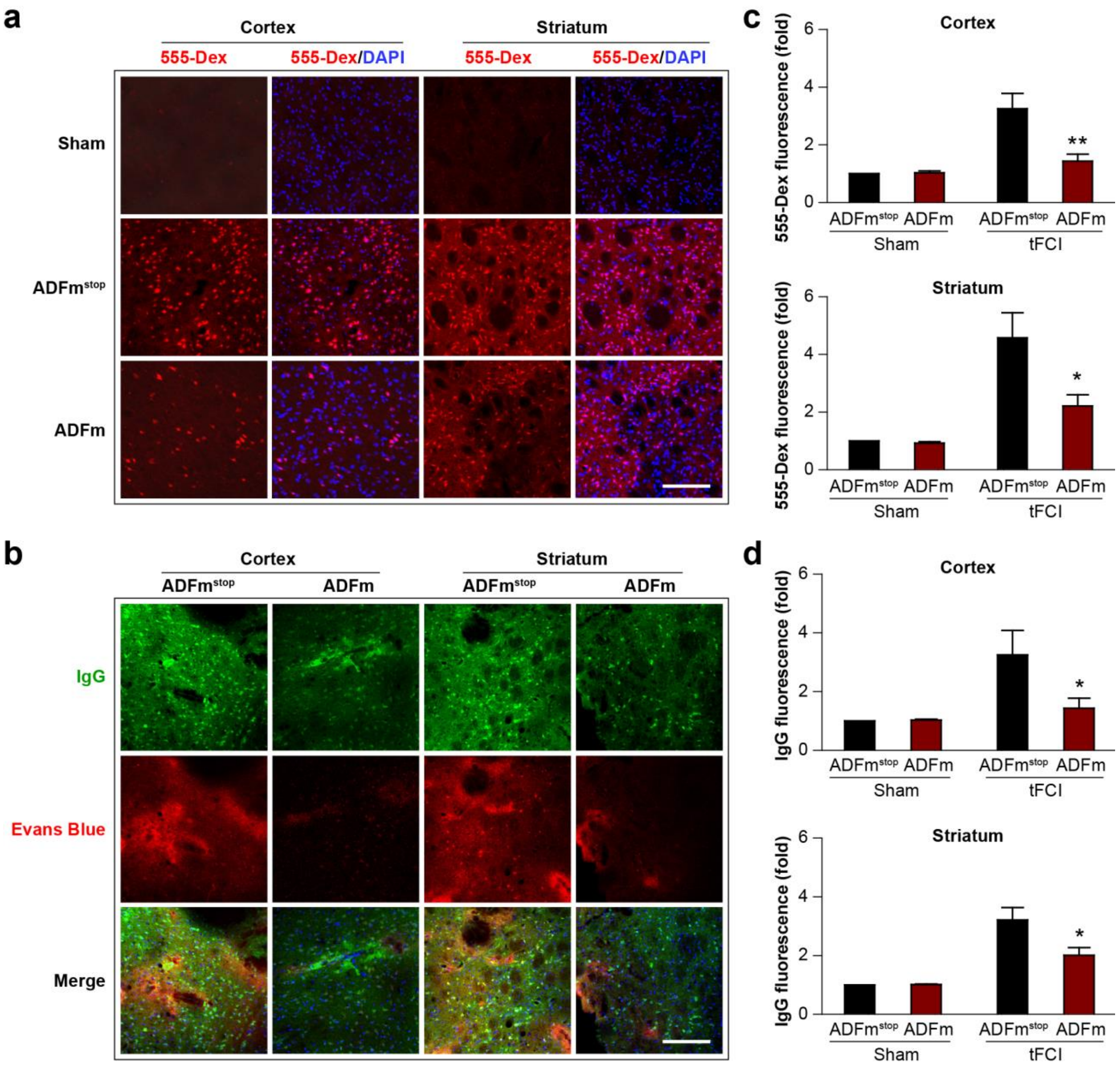

Supplementary Figure 7 | ADFm expression preserves BBB integrity after tFCI.

Uncrossed Tg-ADFm^{stop} mice or Cre-recombined Tg-ADFm mice were subjected to 1 h of tFCI and 1 h (**a**) or 24 h (**b**) of reperfusion. (**a**) Representative microscopic images (magnification: x200) demonstrate the extravasation of Alexa 555-dextran (3 kDa, *red*) into ipsilateral cortical and striatal parenchyma at 1 h after tFCI, which resulted in positive staining of non-vascular cells. Sections were counterstained with DAPI (*blue*) for nuclear labeling. *Scale bar*: 100 µm. EC-targeted expression of ADFm markedly reduced the extravasation of Alexa 555-dextran in both the cortex and striatum. (**b**) Representative images show the leakage of endogenous plasma IgG (*green*) and the injected Evans blue dye (*red*) into ipsilateral cortical and striatal parenchyma at 24 h

after tFCI. *Scale bar*: 100 µm. EC-targeted expression of ADFm attenuated the extravasation of both plasma IgG and Evans blue. (**c,d**) Fluorescence intensity of Alexa 555-dextran and IgG immunostaining was measured, respectively, and expressed relative to the $ADFm^{stop}$ sham group. n=6 mice per group. *$p \leq 0.05$, **$p \leq 0.01$ *versus* $ADFm^{stop}$.

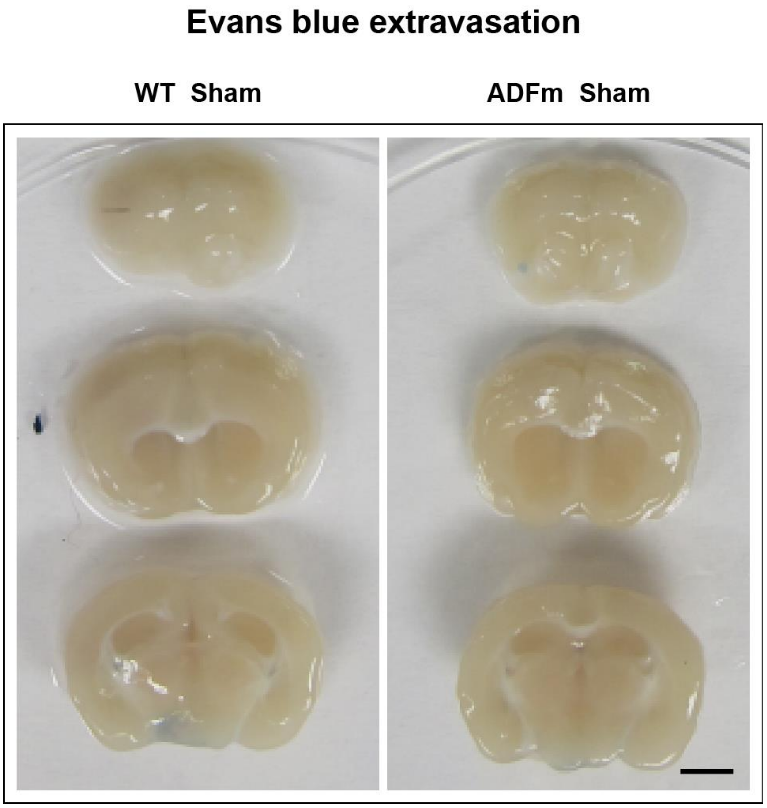

Supplementary Figure 8 | Absence of Evans blue extravasation into the brain parenchyma after sham surgery.

Representative images of coronal brain sections demonstrate that sham operation did not cause leakage of the Evans blue dye into brain parenchyma in either WT or Tg-ADFm animals after 24 h. *Scale bar*: 2 mm.

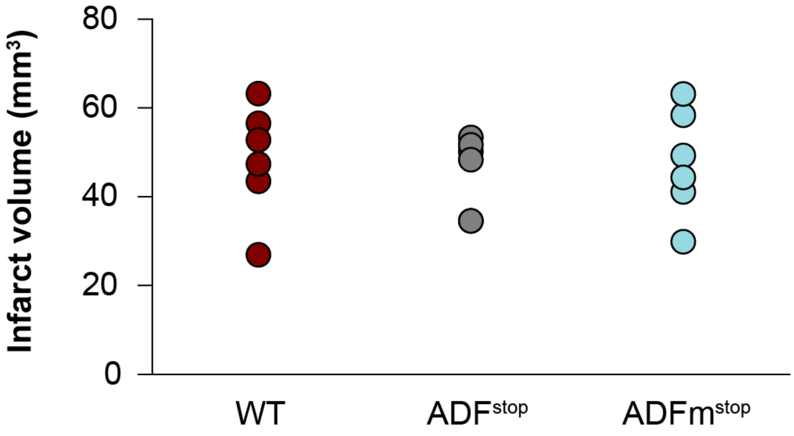

Supplementary Figure 9 | ADF^{stop} or ADFm^{stop} mice exhibit comparable infarct volumes after tFCI compared to WT mice.

Brain infarct volume was measured in TTC-stained slices at 48 h after tFCI in WT and uncrossed Tg-ADF^{stop} or Tg-ADFm^{stop} mice (without crossing with the TEK-Cre mice). No significant difference in infarct volume was observed between any two groups. n=6 mice per group.

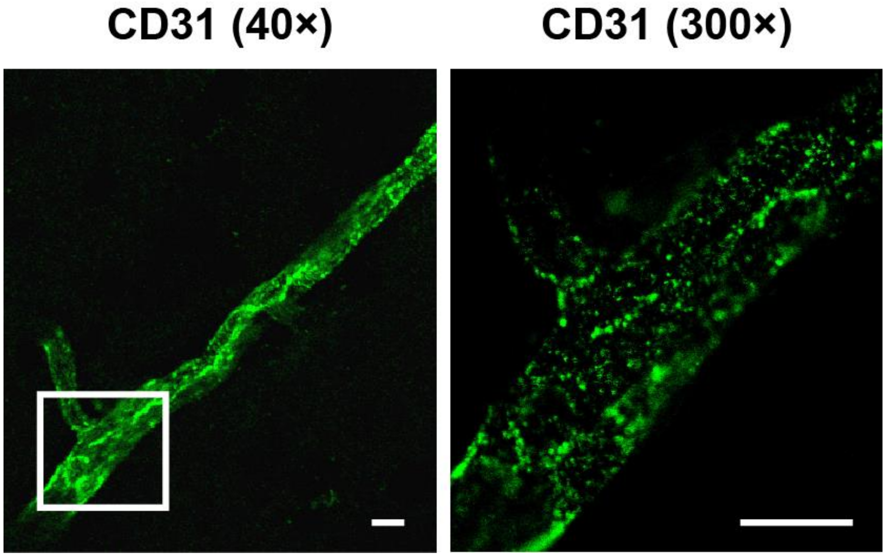

Supplementary Figure 10 | Labeling of brain microvessels with CD31.

Shown are representative images of CD31$^+$ (*green*) microvessels taken under 40× and 300x magnifications, respectively. *Square*: the region enlarged in the high-power image. *Scale bar*: 10 µm. CD31 immunofluorescence shows a relatively diffused pattern under 40×, whereas a punctate pattern was observed in high-power images take under 300×.

Whole-cell

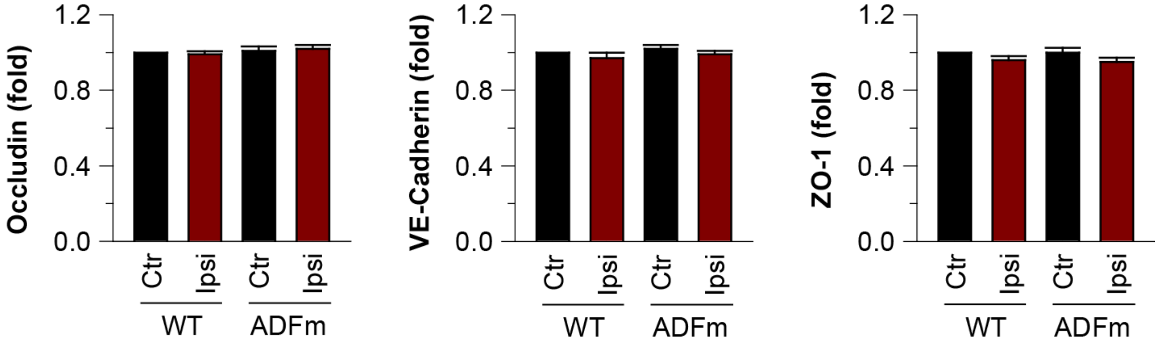

Supplementary Figure 11 | Overexpression of ADFm in ECs does not alter the expression of junctional proteins in whole-cell lysates from brain microvessel extracts.

tFCI was induced for 1 h in WT and Tg-ADFm mice followed by 1 h of reperfusion. Whole cell lysates were prepared from brain microvessel extracts and probed with immunoblotting for occludin, VE-cadherin, ZO-1, and subfraction markers β-actin, CD31, or α-tubulin (see Fig. 7f). Quantification of blots (normalized to WT contralateral) is presented. tFCI does not induce significant loss of junctional proteins at 1 h of reperfusion, nor does endothelial ADFm overexpression alter the level of these proteins in whole-cell microvessel extracts. n=6 mice per group.

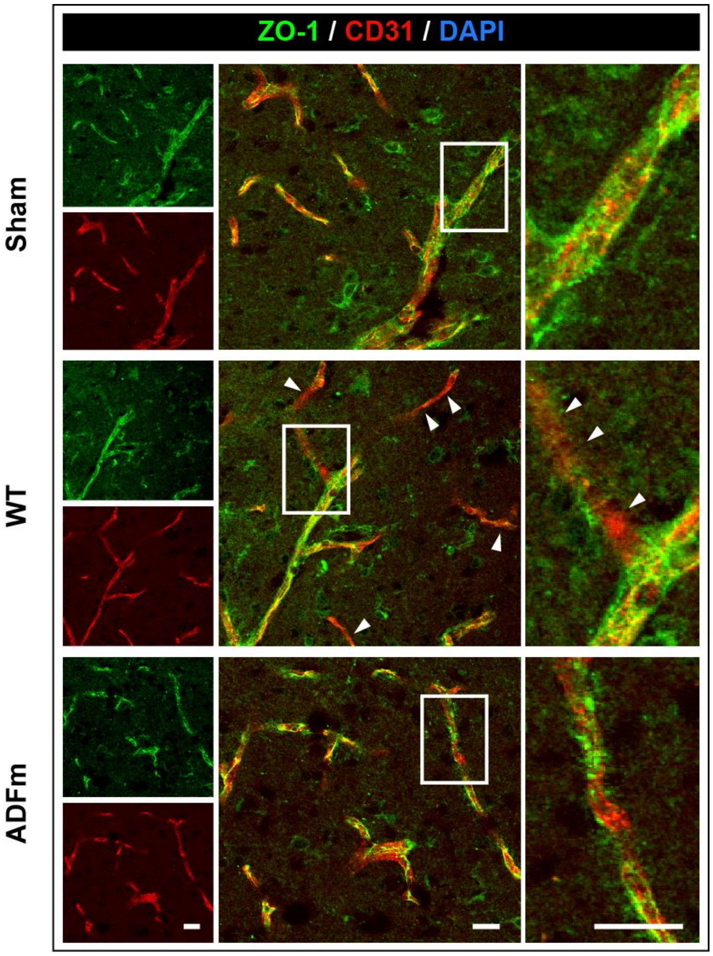

Supplementary Figure 12 | EC-targeted ADFm overexpression reduces ZO-1 degradation after tFCI.

tFCI was induced for 1 h in WT and Tg-ADFm mice followed by 24 h of reperfusion. Double-label immunostaining of the TJ protein ZO-1 (*green*) and endothelial marker CD31 (*red*) in the ischemic cortex is shown. *Scale bar*: 50 µm. In contrast to the distribution pattern of the basal lamina protein laminin, which was observed mainly in the outer layer of $CD31^+$ microvessels (see Fig. 8b), ZO-1 was distributed predominantly inside $CD31^+$ microvessels, as shown by colocalization with CD31 in *yellow*. tFCI caused degradation of ZO-1 protein, demonstrated by the loss of *green* signal and only a *red* color in merged images. ADFm overexpression significantly preserved ZO-1 from tFCI-induced degradation. *Rectangle*: the region enlarged in high power images. *Arrowhead*: loss of ZO-1 immunostaining.

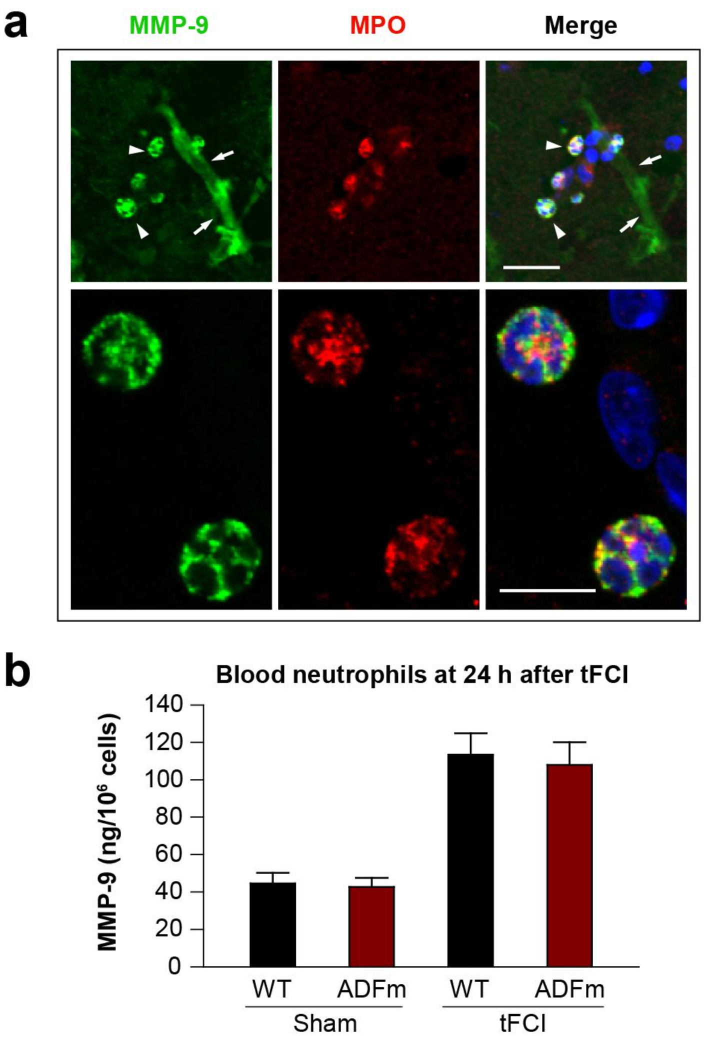

Supplementary Figure 13 | ADFm overexpression does not alter MMP-9 production in blood neutrophils.

tFCI was induced for 1 h in WT and Tg-ADFm animals followed by 24 h of reperfusion. (**a**) Double-label immunostaining for MMP-9 (*green*) and neutrophil marker MPO (*red*) in ipsilateral cortex of WT brains. MMP-9 was expressed in MPO^+ neutrophils (*arrowhead*) and microvessels (*arrow*, see Fig. 8c). *Scale bar*: 50 µm in upper panels and 15 µm in lower panels. (**b**) MMP-9 levels in blood neutrophils were measured by ELISA in WT and Tg-ADFm mice at 24 h after tFCI or sham operation. Endothelial ADFm overexpression did not change neutrophil MMP-9 production. n=6 mice per group.

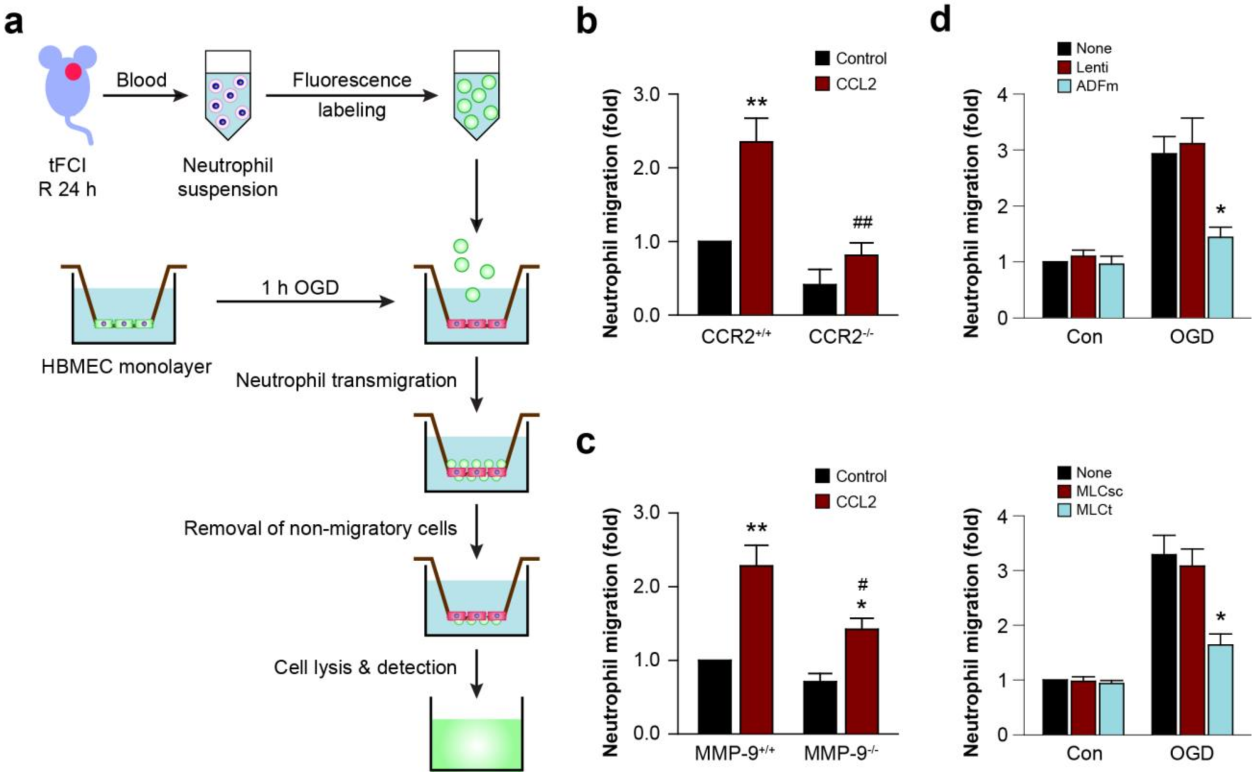

Supplementary Figure 14 | ADFm overexpression reduces neutrophil transmigration across the endothelium after OGD.

(**a**) Illustration of the *in vitro* neutrophil migration assay. Active neutrophils were extracted from the blood of mice at 24 h after tFCI. Neutrophils were labeled with fluorochromes and plated on top of the HBMEC monolayer pre-exposed to 1-h OGD in the migration inserts. Cells were allowed to migrate for 6 h and non-migratory cells were removed. Cells in the bottom chamber were lysed and fluorescence intensity was measured. (**b,c**) Migration of neutrophils obtained from CCR2$^{+/+}$, CCR2$^{-/-}$, MMP-9$^{+/+}$ or MMP-9$^{-/-}$ mice across the HBMEC monolayer was assessed in the presence of absence of 5 ng/mL CCL2 in the bottom chamber. Data were expressed relative to CCR2$^{+/+}$ or MMP-9$^{+/+}$ control with no CCL2. Neutrophil migration was dramatically promoted by CCL2, but hampered in CCR2$^{-/-}$ or MMP-9$^{-/-}$ neutrophils. Data represent 4 independent experiments. *$p \leq 0.05$, **$p \leq 0.01$ *versus* non-CCL2 control. $^{\#}p \leq 0.05$, $^{\#\#}p \leq 0.01$ *versus* CCR2$^{+/+}$ (**b**) or MMP-9$^{+/+}$ (**c**). (**d**) HBMECs were infected with Lenti, Lenti-ADFm, Lenti-MLCsc, or Lenti-MLCt, and subjected to 1 h of OGD. The transmigration of MMP-9$^{+/+}$ neutrophils across the endothelial monolayer was quantified after 6 h of co-culture, and expressed relative to non-transfected non-OGD controls. ADFm overexpression or MLC knockdown in ECs inhibited OGD-induced neutrophil transmigration. Data represent 4 independent experiments. *$p \leq 0.05$ *versus* Lenti or MLCsc.

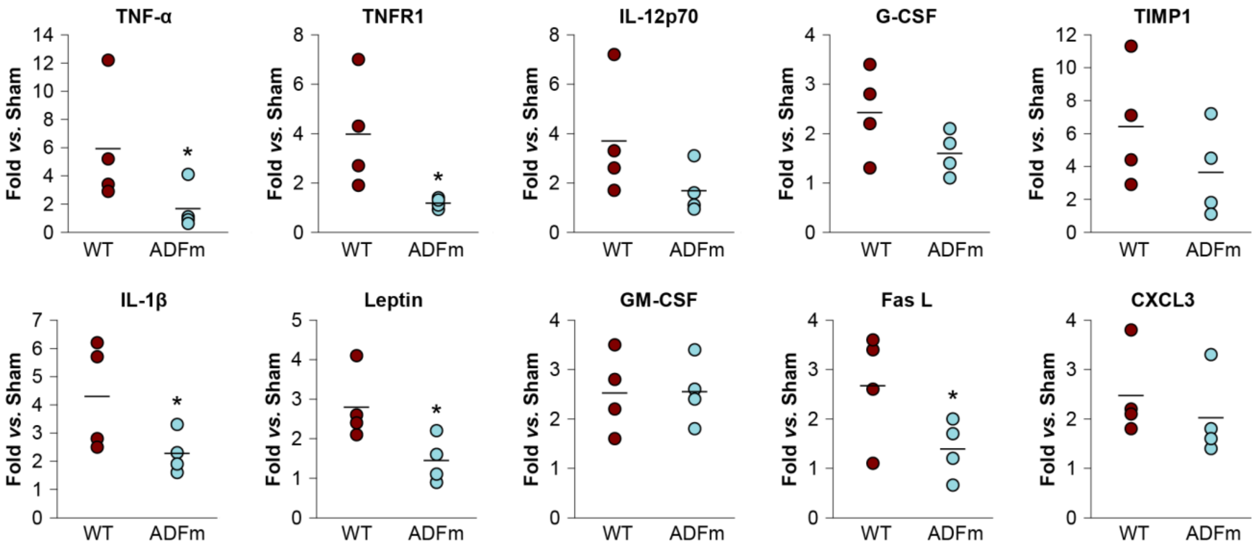

Supplementary Figure 15 | ADFm overexpression reduces the expression of inflammatory markers after tFCI.

A panel of inflammatory markers was examined using the quantitative inflammation array in microvessel extracts from WT and Tg-ADFm brains at 24 h after tFCI. The levels of inflammatory markers were expressed relative to the WT sham group. ADFm overexpression significantly reduced the expression of several markers, including TNF-α, TNFR1, IL-1β, leptin, and FasL. In contrast, no significant changes were observed in IL-12p70, G-CSF, TIMP1, GM-CSF and CXCL3. n=4 mice per group. *$p \leq 0.05$ *versus* WT.

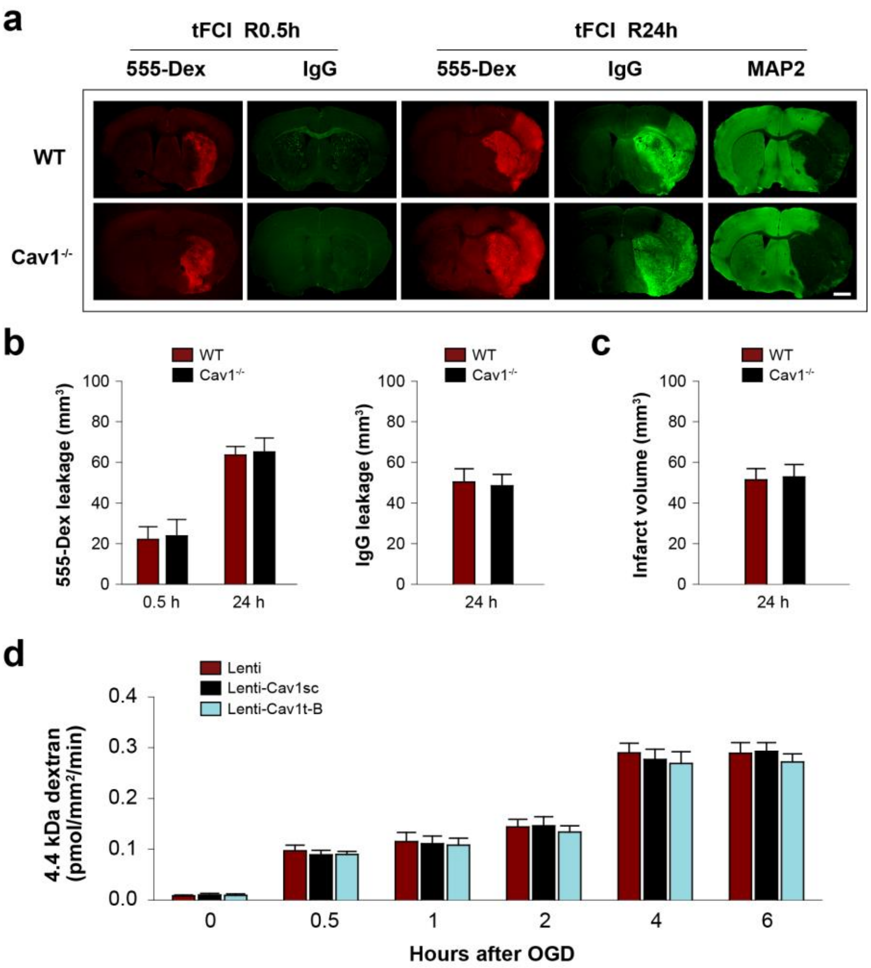

Supplementary Figure 16 | Caveolin1-mediated transcellular mechanisms do not contribute significantly to early BBB disruption after I/R insults.

tFCI was induced in WT and Caveolin1$^{-/-}$ mice for 1 h followed by reperfusion (R). (**a**) Representative images showing the extravasation of Alexa 555-dextran (3 kDa, *red*) or plasma IgG (*green*) into the brain parenchyma after 0.5 or 24 h of reperfusion. *Scale bar*: 1 mm. Loss of MAP2 immunostaining was used to illustrate the infarcts in the same brains after 24 h of reperfusion. (**b**) Volume of leakage of Alexa 555-dextran and endogenous IgGs at indicated reperfusion time points. (**c**) Brain infarct volumes were measured after 24 h of reperfusion on MAP2-stained coronal sections. n=4 mice per group. (**d**) Cultured HBMECs were infected with control empty lentivirus (Lenti), or lentiviral vectors carrying Caveolin1-targeting shRNA (Sequence B) or non-targeting scrambled sequences (Cav1-Sc). Cells were cultured in the *in vitro* BBB model, and subjected to 1 h of OGD. The diffusion coefficient of the 4.4 kDa dextran was measured 0-6 h after OGD. Data represent 4 independent experiments. Knockdown of Caveolin1 does not alter BBB permeability in either the *in vivo* or the *in vitro* I/R models.

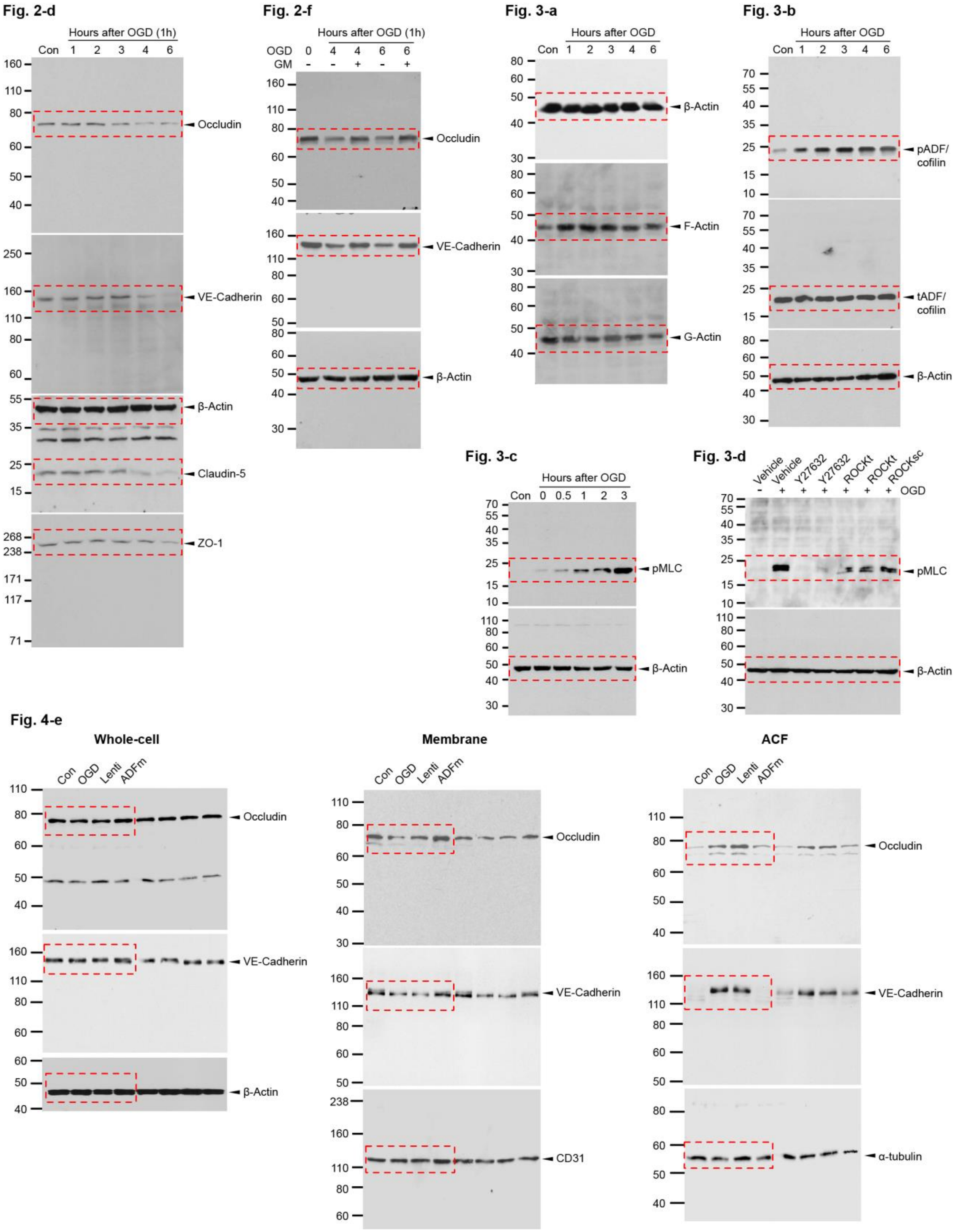

Supplementary Figure 17 | Images of full-length blots presented in the figures.

Fig. 4-f

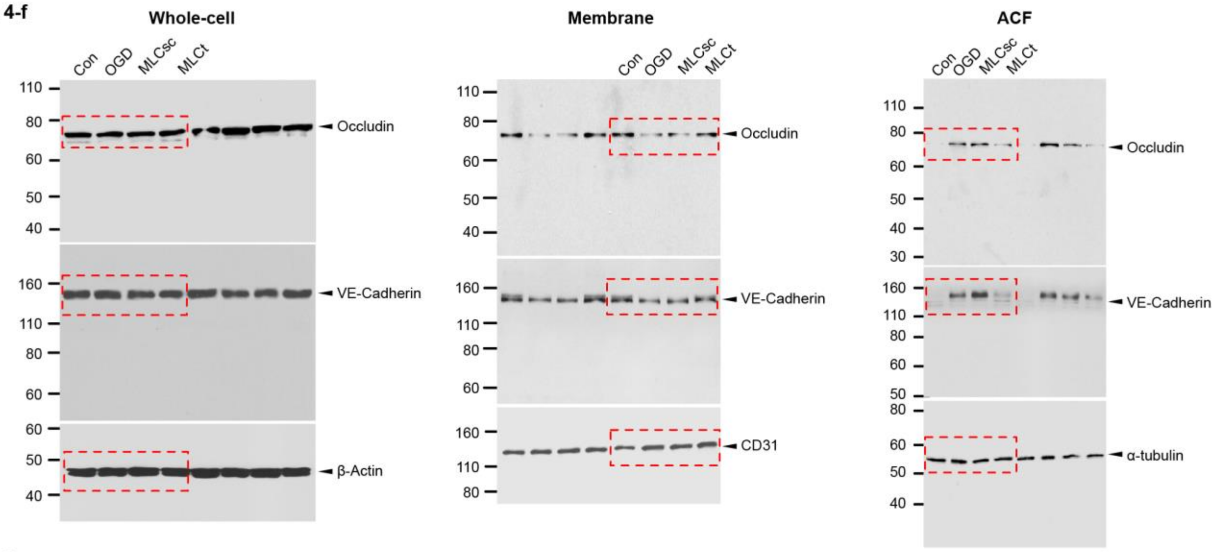

Fig. 7-a

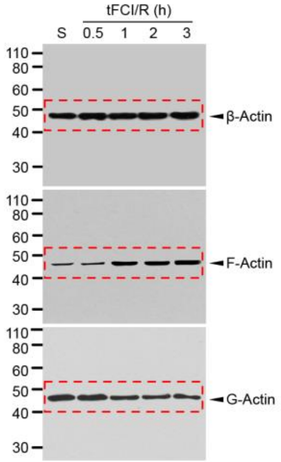

Fig. 7-b

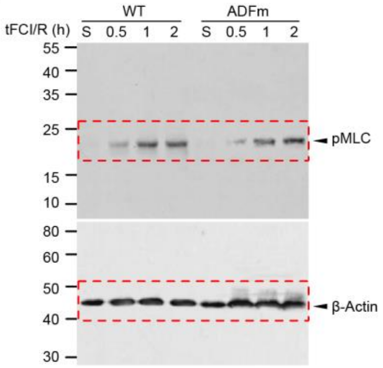

Fig. 7-d

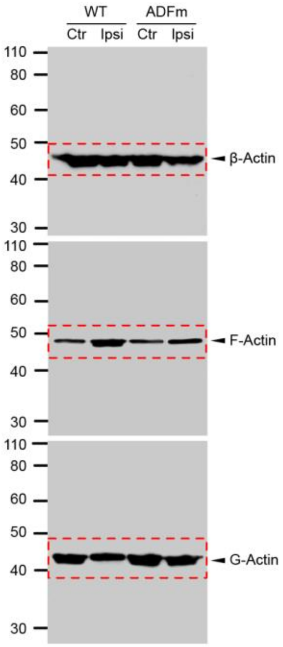

Fig. 7-f

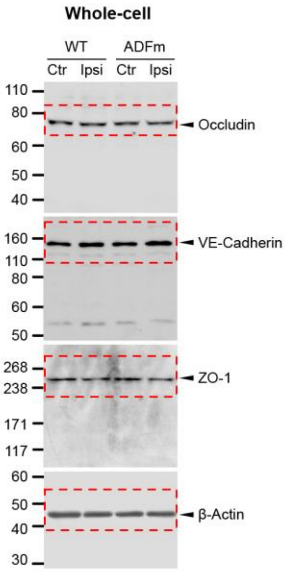

Supplementary Figure 17 (continued) | Images of full-length blots presented in the figures.

Fig. 7-f

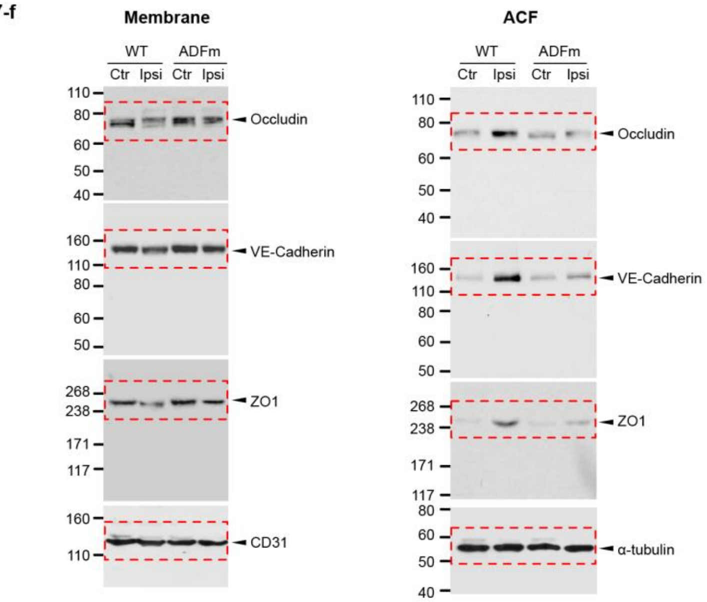

Fig. 8-a

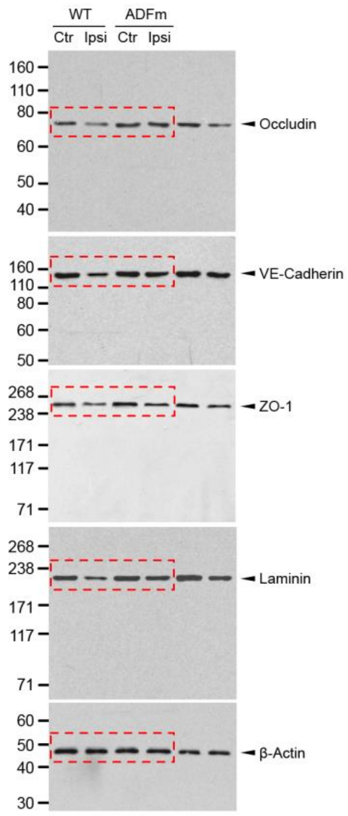

Fig. 8-d

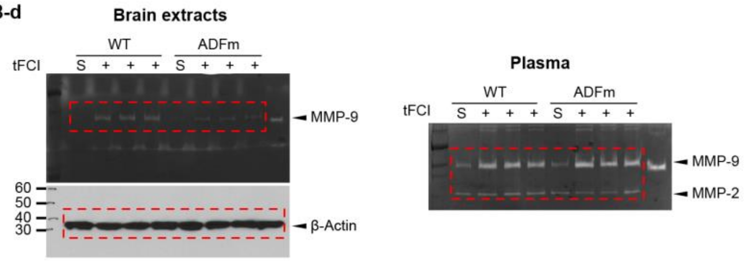

Fig. 8-e

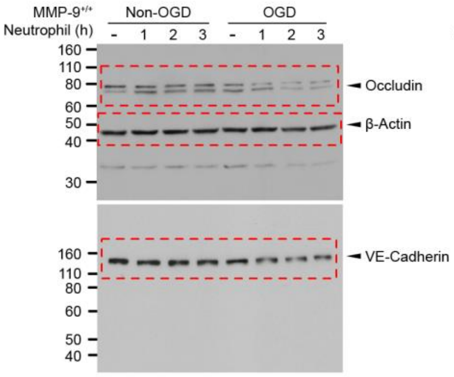

Fig. 8-f

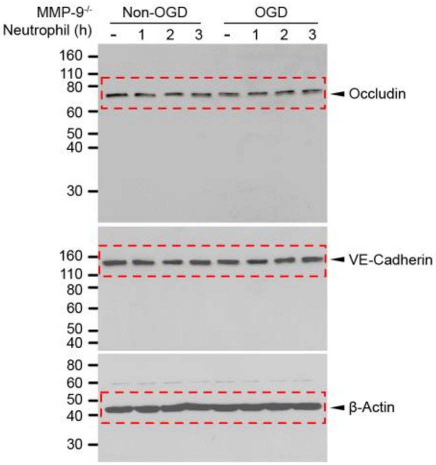

Fig. 8-g

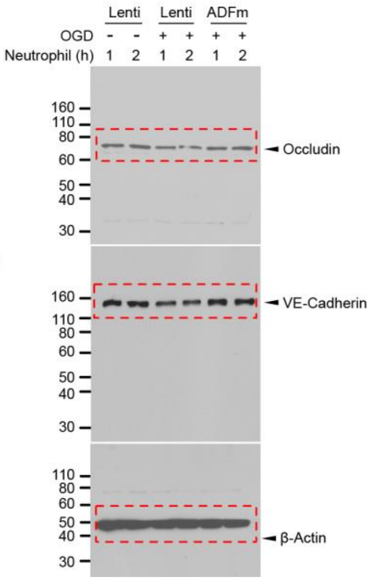

Supplementary Figure 17 (continued) | Images of full-length blots presented in the figures.

Supplementary Fig. 2-a

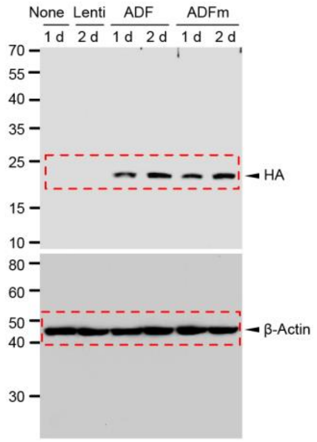

Supplementary Fig. 2-c

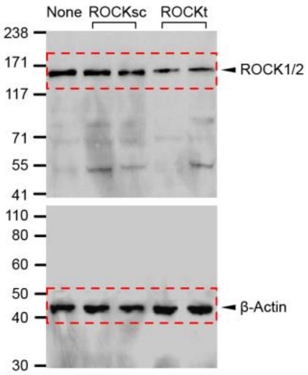

Supplementary Fig. 5-c

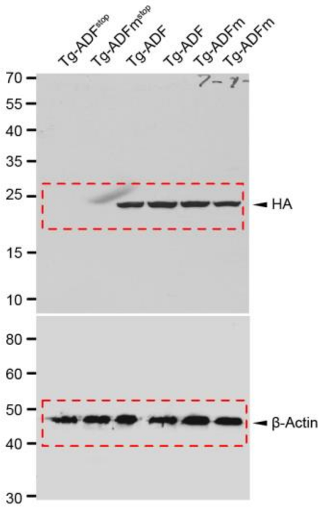

Supplementary Fig. 2-b

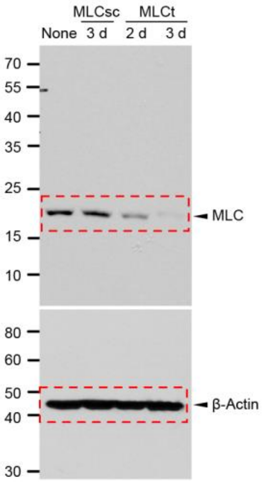

Supplementary Fig. 2-d

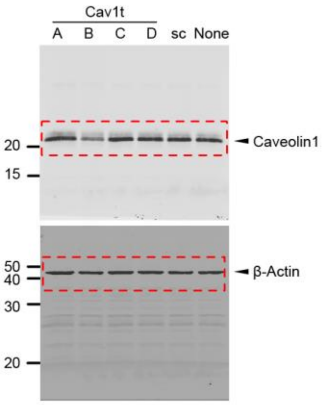

Supplementary Figure 17 (continued) | Images of full-length blots presented in the figures.

Supplementary Table 1 | Regional cerebral blood flow during and after tFCI.

	Time after ischemia*		
	15 min	45 min	R 15 min
WT	12.5 ± 2.5%	12.8 ± 2.6%	78.0 ± 6.4%
MMP-9$^{-/-}$	12.0 ± 2.7%	11.8 ± 2.9%	82.0 ± 6.0%
GM6001	11.0 ± 2.5%	11.4 ± 2.9%	72.0 ± 6.4%

* Regional cerebral blood flow measurements were taken 15 and 45 min after the onset of ischemia, and 15 min after the onset of reperfusion (R). Data are expressed as percentages of baseline levels before ischemia. Values shown are mean ± SEM. n=6 mice per group. No significant difference was observed between WT, MMP-9$^{-/-}$ and GM6001-treated mice.

Supplementary Table 2 | Evaluation of surface cerebral blood vessels in the MCA territory and PcomA plasticity in mice.

	MCA distance from the midline[a] at the specified coronal plane[b] of:			***Number of hemispheres with PcomA scores[c] of:***			
	2 mm	4 mm	6 mm	0	1	2	3
WT	2.24 (0.04)	2.36 (0.06)	2.43 (0.05)	1	6	3	0
Tg-ADF[stop]	2.21 (0.04)	2.29 (0.04)	2.39 (0.04)	1	7	2	0
Tg-ADFm[stop]	2.31 (0.06)	2.40 (0.05)	2.46 (0.06)	2	6	2	0
Tg-ADF	2.26 (0.05)	2.33 (0.04)	2.41 (0.06)	1	6	3	0
Tg-ADFm	2.28 (0.04)	2.41 (0.05)	2.44 (0.07)	0	7	3	0

[a] Values shown are mean (SEM) in millimeters (n=5 mice per group).

[b] A coronal plane is specified based on its distance from the frontal pole in millimeters.

[c] PcomA (posterior communicating artery) plasticity scoring criteria: 0, no anastomosis between PCA and SCA; 1, anastomoses between PCA and SCA in capillary phase; 2, small truncal anastomoses between PCA and SCA; 3, truncal anastomoses between PCA and SCA.

Supplementary Table 3 | Physiological parameters during and after tFCI.

	Time*	pH	pO_2	pCO_2	Glucose
WT (6)	During tFCI	7.36 ± 0.02	129.10 ± 2.70	41.80 ± 1.06	121.4 ± 2.4
	After tFCI	7.34 ± 0.02	132.40 ± 2.27	42.70 ± 1.17	136.1 ± 3.0
Tg-ADFm^{stop} (6)	During tFCI	7.38 ± 0.02	124.80 ± 3.10	42.20 ± 0.78	126.6 ± 3.8
	After tFCI	7.33 ± 0.03	134.10 ± 1.68	42.20 ± 1.84	139.0 ± 3.1
Tg-ADF (8)	During tFCI	7.36 ± 0.01	128.30 ± 3.30	41.40 ± 1.29	118.8 ± 3.1
	After tFCI	7.35 ± 0.01	130.60 ± 2.24	41.90 ± 1.23	134.3 ± 3.4
Tg-ADFm (8)	During tFCI	7.35 ± 0.01	131.00 ± 4.05	41.80 ± 1.25	128.5 ± 3.6
	After tFCI	7.34 ± 0.02	129.30 ± 3.09	42.80 ± 1.33	140.8 ± 3.3

* Measurements were taken 15 min after the onset of tFCI (during tFCI) and 15 min after the onset of reperfusion (after tFCI). Physiological parameters measured are: pO_2 (arterial O_2 pressure; mmHg), pCO_2 (arterial CO_2 pressure; mmHg), and glucose (blood glucose level; mg dL^{-1}). Values shown are mean ± SEM. The number of mice in each group is shown in parentheses. No significant difference was observed across genotype in any of the parameters.

Supplementary Table 4 | Regional cortical blood flow during and after tFCI.

	**Time after ischemia***		
	5 min	15 min	R 15 min
WT (6)	12.2 ± 1.6%	13.8 ± 1.4%	78.8 ± 2.1%
Tg-ADFm^{stop} (6)	11.8 ± 1.4%	14.2 ± 1.3%	80.4 ± 2.8%
Tg-ADF (8)	12.3 ± 1.5%	14.4 ± 1.9%	81.3 ± 2.5%
Tg-ADFm (8)	12.6 ± 1.3%	14.5 ± 1.7%	79.8 ± 2.8%

* Measurements were taken 5 min and 15 min after the onset of ischemia and 15 min after the onset of reperfusion (R). Data are expressed as percentages of pre-ischemia baseline levels. Values shown are mean ± SEM. The number of mice in each group is shown in parentheses. No significant difference across genotype was found.

Supplementary Methods

As mentioned in the main text, *Stroke Therapy Academic Industry Roundtable (STAIR)* guidelines[1] were strictly followed throughout the experiments. For example, animals were randomly selected for experiments with a lottery-drawing box. We verified that blood pH, gases, glucose levels, and cerebral blood flow were not altered by transgene expression. Furthermore, surgeries and all outcome assessments were performed by investigators blinded to mouse genotype and experimental group assignments.

Generation of Tg-ADFm^{stop} or Tg-ADF^{stop} mice for endothelial cell-targeted overexpression of actin depolymerizing factor mutant (ADFm) or ADF

Tg-ADFm^{stop} or Tg-ADF^{stop} transgenic mice were generated for conditional overexpression of ADFm and ADF, respectively. Briefly, a targeting vector was constructed to contain WT human ADF or its constitutively active mutant (S3A, ADFm) downstream of a *loxP*-flanked stop sequence (neomycin resistance gene and a trimer of the SV40 polyadenylation sequence). This construct was inserted into the *Gt(ROSA)26Sor* locus *via* electroporation of embryonic stem (ES) cells, and the correctly targeted ES cells were subjected to microinjection in C57BL/6J blastocysts. The ES cell screening and microinjection procedures were performed at the Shanghai Research Center for Model Organisms through a service contract. Mutant mice progeny were backcrossed to the C57/B6 background for at least six generations before use to minimize the potential influence of genetic heterogeneity on the susceptibility of animals to cerebral ischemia. To obtain EC-specific ADF- or ADFm-overexpressing mice, homozygous Tg-ADF^{stop} or Tg-ADFm^{stop} mice were crossed with Tek-Cre mice[2], in which the Cre recombinase expression is driven by the *Tek* (endothelial-specific receptor tyrosine kinase) promoter and thus restricted to ECs. In the presence of Cre recombinase, the stop codon is excised and a 5’ HA-tagged ADF or ADFm protein is expressed specifically in ECs (Tg-ADF or Tg-ADFm). Overexpression of ADF/ADFm was validated by immunohistochemistry and Western blotting for HA (see Supplementary Fig. 6).

Choose of sample size

The number of animals required for the *in vivo* studies was determined by power analysis based on our experience with the murine MCAO model. To detect a 30% decrease in infarct volume or neurological deficits with 80% power at an α value of 0.05 (two-tailed), approximately 6-8 mice per group was needed. For blood-brain transfer coefficient and immunohistochemistry, 4 samples were required for 80% power (β=0.8, α=0.05) to detect a 30% change after tFCI. For Western blotting and ELISA analysis, 4-

5 samples were required for 80% power (β=0.8, α=0.05) to detect a 30% change. Brains from 2 mice needed to be pooled together to prepare 1 sample of single cell suspension for flow cytometry analysis. Four samples (8 mouse brains) are required for 80% power (β=0.8, α=0.05) to detect a 30% change after tFCI.

Evaluation of mouse cerebrovascular anatomy

Cerebrovascular anatomy was quantitatively evaluated in Tg mice and their WT littermates as we described before[3], to assess the potential impact of variations in the anatomy of the cerebral circulation on susceptibility to ischemic injury. Briefly, mice were sacrificed by CO_2 overdose, and transcardial perfusion fixation was performed immediately *via* the left ventricle with heparinized saline (10 units per mL) followed by warm formalin. Evans blue (EB; Sigma-Aldrich; 2% in normal saline, with sonication) was mixed with gelatin (20% in water) in equal volumes and kept warm to prevent solidification. The mixture was then injected through a cannula into the ascending aorta. Brains were harvested and stored in formalin. Ventral and dorsal photographs of the brain were taken with a dissecting microscope to visualize the middle cerebral artery (MCA) and posterior cerebral artery (PCA) territories. The MCA territory was determined by the localization of anastomoses, and distances between the midline and the anastomoses were measured at coronal planes 2, 4, and 6 mm from the frontal pole. The plasticity of the posterior communicating artery (PcomA) was graded on a qualitative scale of 0-3: 0, no anastomosis between PCA and superior cerebellar artery (SCA); 1, anastomoses between PCA and SCA in capillary phase; 2, small truncal PcomA; 3, truncal PcomA.

Vascular labeling and three-dimensional analysis of vascular density

Brain microvessels were labeled by perfusion with lectin, as we described previously[4]. Briefly, mice were transcardially perfused with FITC-conjugated tomato lectin (Sigma-Aldrich) at a dose of 100 µg μL^{-1}. Coronal brain sections were prepared and imaged as described in *Methods*. Six sections, 0.5-mm apart, were analyzed for each brain, and six regions of interest (ROIs; 233 × 233 μm^2) in the cerebral cortex or striatum were selected from each section. The ROIs were scanned at 512 × 512 pixels in the x-y direction, and 1-µm-step-size optical sections along the z-axis were acquired with a 40× objective lens. Three-dimensional reconstruction was performed using an image analysis software package (3D Doctor 3.5, Able software, USA). The vascular surface area (mm^2) and the total vascular length (mm) per volume of tissue (mm^3) were calculated by the software, and the number of vascular branch points was counted in the three-dimensional images by a blinded investigator.

Two-dimensional laser speckle imaging

Cortical cerebral blood flow (CBF) was monitored using the laser speckle technique as we described previously[5]. Briefly, a charge-coupled device camera (PeriCam PSI System; Perimed Inc., Ardmore, PA, USA) was placed above the head, and the intact skull surface was illuminated by a laser diode (785 nm) to allow laser penetration through the brain in a diffuse manner. Speckle contrast, defined as the ratio of the standard deviation of pixel intensity to the mean pixel intensity, represents the speckle visibility relative to the velocity of the light-scattering particles (blood) and was therefore used to measure cortical blood flow. The speckle contrast was then converted to correlation time values, which were inversely proportional to the mean blood flow velocity. Two-dimensional microcirculation images were obtained 15 min before tFCI and continued throughout the ischemic period until 15 min after the onset of reperfusion. The area of the ischemic core (0-20% residual CBF) or the penumbra (20-30% residual CBF) region[6, 7] was measured from laser speckle images.

Neurobehavioral tests

Neurobehavioral tests were carried out 1 d before and 1-28 d after MCAO. Sensorimotor deficits were evaluated by the rotarod, cylinder and corner tests. Long-term cognitive deficits were evaluated by the Morris water maze test.

Rotarod test

The rotarod test was performed to assess post-stroke motor functions as we described previously[6]. Briefly, animals were placed on a rotating drum with a speed accelerating from 2.5 to 25 rpm within 5 min. The time at which the mouse fell off the drum (latency to fall) was recorded. The test began 1 d before surgery and consisted of 2 trials. On the day of surgery, 5 trials were performed on each mouse and the mean of trial # 3, 4, and 5 was used as the pre-surgery baseline value. After surgery, mice were tested for 5 trials *per* day with intervals of 15 min, and the data for trial #3-5 were expressed as the mean latency to fall on each testing day.

Cylinder test

The cylinder test was performed to assess forepaw use asymmetry, as we described previously[6]. The mouse was placed in a transparent cylinder (diameter: 9 cm; height: 15 cm), and videotaped for 5 min. A mirror was placed behind the cylinder at an angle which allowed the rater to record all forepaw movements. Videotapes were analyzed in slow motion, and forepaw (left/right/both) use during the first contact against the cylinder wall after rearing and during lateral exploration was recorded. Preference of the non-

impaired forepaw (left) was calculated as a relative proportion of right forepaw contacts: (left-right)/(left+right+both)×100%. Uninjured mice typically show no preference for either forepaw, whereas injured mice have increased left forepaw preference depending on the severity of the injury.

Corner test

The corner test was performed as we described previously[8]. The injured animal turns preferentially toward the non-impaired (left) side. Performance was expressed as the number of left turns out of 10 trials for each test.

Morris water maze test

The Morris water maze test was carried out 23-28 d after tFCI to evaluate long-term cognitive functions, as we described previously[9]. Briefly, a platform (diameter: 11 cm) was submerged in a pool (diameter: 109 cm) of opaque water. Mice were placed into the pool from one of the four locations and allowed 90 s to locate the hidden platform (learning phase of the test). The time at which the animal found the platform (escape latency) was recorded for each trial. At the end of each trial, the mouse was placed on the platform or allowed to stay on the platform for 30 s with prominent spatial cues displayed around the room. Four trials were performed on each day for 5 consecutive days. After the last day of the hidden platform test, a single, 60-s probe trial was performed in which the platform was removed. The time spent in the target quadrant where the platform was previously located was recorded (memory phase of the test). Swim speed was also recorded to assess locomotor function.

Measurement of albumin leakage

BBB permeability and leakage of plasma albumin was determined by measuring EB extravasation as we described previously[10]. Briefly, 2.5% EB (5 mL kg^{-1}) was injected into the femoral vein. Three hours later, animals were transcardially perfused with cold saline to remove intravascular EB. Coronal brains sections (2 mm thick) were cut and EB extravasation was visualized. Alternatively, EB fluorescence was examined on 30-µm coronal sections using confocal microscopy, as described in *Methods*. To quantify EB extravasation, sections were carefully weighed and soaked in methanamide for 48 h at 37.0°C, and subsequently centrifuged for 30 min at 14,000 rpm. The absorption of the supernatant was measured at 632 nm with a spectrophotometer. EB concentration in the tissue was quantified using a standard curve and expressed as ng per mg of protein.

Measurement of infarct volume

Forty-eight hours after tFCI, mice were sacrificed and brains were harvested. The forebrain was sliced into 7 coronal sections, each 1 mm thick. Sections were stained with 3% 2,3,5-triphenyltetrazolium (TTC) in saline for 20 min, followed by fixation with 4% paraformaldehyde in PBS, pH 7.4. Infarct volume was determined using MCID image analysis[8] by an observer blinded to experimental group assignment.

F-actin/G-actin assay

Actin polymerization in HBMEC cultures and brain microvessels was evaluated using the G-actin/F-actin In Vivo Assay Biochem Kit (Cytoskeleton, Inc., Denver, CO, USA) according to manufacturer's instructions. Briefly, lysate were collected from cultured HBMECs or isolated brain microvessels using the Lysis and F-actin Stabilization Buffer provided in the kit. F-actin and G-actin were separated by centrifugation at 100,000 × *g* for 1 h at 37°C. The supernatant containing G-actin was collected, and the F-actin in the pellets was depolymerized to the globular form by the F-actin Depolymerization Buffer. Samples from both the G- and F-actin components were examined by Western blotting with anti-actin antibodies as described in *Methods*. The ratio of F-actin/G-actin was measured by densitometry as an index of actin polymerization.

Lentiviral vectors for gene overexpression or knockdown

Lentiviral vectors were constructed overexpressing human full-length ADF (Lenti-ADF) or its constitutively active form bearing single amino acid substitution (S3A; Lenti-ADFm), as we described previously[8]. The HA-tagged cDNA was inserted into the lentiviral transfer vector FSW under the control of the Tie-2 promoter. The constructed transfer vectors were transformed into Stbl3 *Escherichia coli*, and then isolated using the EndoFree Plasmid Maxi Kit (Qiagen, Valencia, CA, USA). Large-scale production of the virus was achieved as we described previously[8]. Briefly, a plasmid mixture containing 435 µg of pCMV ΔR8.9 (packaging construct), 237 µg of pVSVG (envelope plasmid), and 675 µg of FSW (transfer vector) was suspended in 34.2 mL of $CaCl_2$ (250 mM) and then added volume for volume into 2× BES buffer, pH 6.95. The DNA-$CaCl_2$ precipitate was added to human kidney 293 FT cells (on 15-cm plates at a density of 1.1×10^7 per plate) drop by drop (1.125 mL for each plate), and allowed to incubate for 12 h before switching to fresh culture medium. The supernatant was collected 72 h after transfection, filtered through the 0.45 µm filter flask and centrifuged at 21,000 rpm for 2 h using the SW28 rotor (Beckman Coulter). Viruses were further purified by sucrose gradient ultracentrifugation. The pellet was suspended in 3 mL of PBS, loaded on the top of 2 mL of 20% sucrose solution, and centrifuged at 22,000 rpm for 2 h using the SW50.1 rotor (Beckman Coulter). The resulting pellet was

resuspended in 200 µL of DMEM, aliquoted, and stored at -70°C. The titer of the vector stock was determined using enzyme-linked immunosorbent assay (ELISA). The average titer is typically ∼5-10 × 10^{10} particle units per mL.

Lentiviral vectors expressing short hairpin interfering RNA (shRNA) against murine MLC or ROCK, or its counterpart scrambled sequence (MLCsc or ROCKsc) were purchased from Santa Cruz. Caveolin1 shRNA was purchased from OriGene. The HBMEC cultures were infected with Lenti-ADF, Lenti-ADFm, Lenti-MLCt, Lenti-ROCKt, Lenti-Cav1t, Lenti-MLCsc, Lenti-ROCKsc, Lenti-Cav1sc, or the control empty vector. The overexpression of ADF/ADFm, or knockdown of MLC, ROCK, or Caveolin1 in HBMECs was confirmed by Western blot analyses. Experiments were performed 48 h (for overexpression) or 72 h (for knockdown) after transfection.

In vitro neutrophil migration assay

Neutrophil transmigration across the BBB was measured *in vitro* (see Supplementary Fig. 14) using the CytoSelect™ Leukocyte Transmigration Assay kit (Cell Biolabs, Inc., San Diego, CA, USA) according to the manufacturer's instructions. Briefly, an HBMEC monolayer was grown to confluence in the migration insert provided in the kit and subjected to 1-h OGD. Blood neutrophils were extracted from post-tFCI mice as described in *Methods*. Neutrophils were labeled with the fluorochrome LeukoTracker™ (Cell Biolabs), and plated in the upper chamber of the migration insert. Neutrophils were allowed to migrate for 6 h and non-migratory cells were removed. The migration of labeled neutrophils from the upper to the lower chamber was quantified by measuring the fluorescence intensity of cell lysates collected from the lower chamber. Fluorescence was measured with a spectrofluorometer at 480 nm/520 nm and expressed as relative fluorescence units (RFU).

Zymography

Twenty-four hours after tFCI, brain and blood samples were collected for gel zymography as we described previously[11]. Briefly, anti-coagulated blood samples were centrifuged at 4000 rpm for 15 min, and plasma was collected and frozen at -80°C until use. Brain samples were homogenized in lysis buffer (50 mM Tris-HCl, 150 mM NaCl, 5 mM $CaCl_2$, 0.05% BRIJ-35, 0.02% NaN_3, 1% Triton X-100, pH 7.6), and then centrifuged to obtain supernatants. Total protein concentrations were determined (Bio-Rad, Hercules, CA, USA). Forty mg of total protein, which was corrected for protein concentration, 5 µL plasma, and 1 µg of MMP-9 human standards (EMD Millipore) were diluted in an equal volume of 2× non-reducing sample buffer (0.4 M Tris, 5% SDS, 20% glycerol, 0.05% bromophenol blue, pH 6.8). Samples were loaded and separated by a 10% Tris-glycine gel with 0.1% gelatin, and then washed with renaturing buffer

(Invitrogen) for 90 min and further incubated in developing buffer (Invitrogen) at 37°C for 30 h. The gels were then stained with 0.5% Coomassie blue R-250 (Bio-Rad) for 1 h and destained with destain buffer (Bio-Rad). Band intensities of pro- and active-MMP-9 were quantified using the gel analysis function in ImageJ software and expressed as a fold change over sham group.

ELISA

Neutrophil-derived MMP-9 was measured by ELISA as we described previously[11]. Briefly, blood neutrophils were isolated from post-tFCI mice as described in *Methods*. Cells were counted using a haemocytometer, and lysed in lysis buffer (Cell Signaling). Cell lysates were subjected to MMP-9 ELISA according to manufacturer's instructions (R&D System).

Cell viability assays

Cultured HBMECs were assessed for metabolic viability after OGD, using the MTT assay. Cells were incubated with 0.5 mg mL^{-1} of MTT at 37°C for 2 h. Medium was then carefully removed and 100 µL of dimethylformamide was added into each for 1 h well to dissolve the resulting dark blue crystals. Absorbance was measured at 570 nm (OD 570) with a Universal Microplate Reader (Elx800, BioTek Instruments, Winooski, VT, USA). Alternatively, cell damage was evaluated using the LDH assay for loss of membrane integrity. The activity of LDH released into the medium was assayed spectrophotometrically by monitoring the reduction of NAD^+ at 340 nm at 25°C in the presence of lactate (Pointe Scientific, Lincoln Park, MI, USA). Data was expressed as percentage of the maximum LDH activity of the total lysed cells.

Supplementary References

1. Fisher, M. *et al.* Update of the stroke therapy academic industry roundtable preclinical recommendations. *Stroke* **40**, 2244-2250 (2009).

2. Koni, P. A. *et al.* Conditional vascular cell adhesion molecule 1 deletion in mice: impaired lymphocyte migration to bone marrow. *J. Exp. Med.* **193**, 741-754 (2001).

3. Yin, X. M. *et al.* Bid-mediated mitochondrial pathway is critical to ischemic neuronal apoptosis and focal cerebral ischemia. *J. Biol. Chem.* **277**, 42074-42081 (2002).

4. Wang, J. *et al.* Omega-3 polyunsaturated fatty acids enhance cerebral angiogenesis and provide long-term protection after stroke. *Neurobiol. Dis.* **68**, 91-103 (2014).

5. Li, P. *et al.* Adoptive regulatory T-cell therapy preserves systemic immune homeostasis after cerebral ischemia. *Stroke* **44**, 3509-3515 (2013).

6. Gan, Y. *et al.* Transgenic overexpression of peroxiredoxin-2 attenuates ischemic neuronal injury via suppression of a redox-sensitive pro-death signaling pathway. *Antioxid. Redox. Signal.* **17**, 719-732 (2012).

7. Leak, R. K. *et al.* HSP27 protects the blood-brain barrier against ischemia-induced loss of integrity. *CNS Neurol. Disord. Drug Targets* **12**, 325-337 (2013).

8. Stetler, R. A. *et al.* Hsp27 protects against ischemic brain injury via attenuation of a novel stress-response cascade upstream of mitochondrial cell death signaling. *J. Neurosci.* **28**, 13038-13055 (2008).

9. Wang, G. *et al.* Scriptaid, a novel histone deacetylase inhibitor, protects against traumatic brain injury via modulation of PTEN and AKT pathway : scriptaid protects against TBI via AKT. *Neurotherapeutics* **10**, 124-142 (2013).

10. Yu, Q. *et al.* Sevoflurane preconditioning protects blood-brain-barrier against brain ischemia. *Front. Biosci. (Elite Ed)* **3**, 978-988 (2011).

11. Li, P. *et al.* Adoptive regulatory T-cell therapy protects against cerebral ischemia. *Ann. Neurol.* **74**, 458-471 (2013).
